# Integrated liver and serum proteomics uncover sexual dimorphism and alteration of several immune response proteins in an aging Werner syndrome mouse model

**DOI:** 10.18632/aging.205866

**Published:** 2024-05-24

**Authors:** Lucie Aumailley, Marie Julie Dubois, André Marette, Michel Lebel

**Affiliations:** 1 Centre de recherche du CHU de Québec, Faculty of Medicine, Université Laval, Québec City G1V 4G2, Canada; 2 Quebec Heart and Lung Institute, Faculty of Medicine, Université Laval, Québec City G1V 0A6, Canada

**Keywords:** proteomics, Werner syndrome, fatty liver, sexual dimorphism, immunoglobulins

## Abstract

Werner syndrome (WS) is a progeroid disorder caused by mutations in a protein containing both a DNA exonuclease and DNA helicase domains. Previous studies indicated that males lacking the helicase domain of the Wrn protein orthologue exhibited hepatic transcriptomic and metabolic alterations. In this study, we used a label-free liquid chromatography-tandem mass spectrometry approach to uncover proteins abundance associated with specific biological processes that differed depending on the age (four or ten months) and/or the genotype (wild type or Wrn mutant) in the serum and liver of mice. Principal component analysis of the proteomic data from both serum and hepatic tissue revealed a sexual dimorphism regardless of the age and the genotype of the mice. Moreover, although all Wrn mutant mice exhibited fatty liver by the age of ten months, a significant age and genotype dependent enrichment of proteins involved in lipid and fatty acid metabolic processes were uncovered only in males. Also, a genotype dependent increase in serum oxidant detoxification processes was observed in the serum of Wrn mutant males. Despite these sexual differences, several aspects of the immune system were affected in both females and males. Finally, an increase of specific immunoglobulin molecules was common in the liver and serum of both older Wrn mutant females and males. Such results suggest that specific immunoglobulin variants maybe associated with fatty liver progression in WS.

## INTRODUCTION

Aging is a progressive loss of homeostasis leading to an increased susceptibility to diseases. A recurrent theme associated with aging processes is an altered cellular redox status or oxidative stress [1]. A chronic pro-inflammatory status is also a pervasive feature of aging. This chronic, low-grade, systemic inflammation occurring in the absence of overt infection has been defined as “inflammaging” and represents a significant risk factor for morbidity and mortality in the elderly [1]. Interestingly, some specific inherited mutations appear to modulate multiple aspects of aging. These inherited single mutated gene diseases are known as segmental progeroid syndromes. Amongst them, Werner syndrome (WS) is a recessive disorder characterized by the premature onset of several age-related diseases including dyslipidemia, diabetes mellitus, atherosclerosis, and cancer [2]. The mutated gene responsible for WS (*WRN*) codes for a protein containing both a 3′-5′ exonuclease domain and 3′-5′ DNA helicase activity. This protein is involved in transcription, telomere maintenance, DNA repair, and replication [3, 4].

Importantly, WS subjects exhibit oxidative stress and a low but chronic systemic inflammatory phenotype [5, 6]. In addition to metabolic anomalies, individuals with WS have a high prevalence of nonalcoholic fatty liver disease (NAFLD) [7–9]. The liver plays pivotal roles in several detoxification and metabolic processes that impact on normal homeostasis and disease susceptibility to the rest of the body with age. For example, changes in the aging liver will alter the metabolism and the levels of circulating glucose, cholesterol, triglycerides, and lipoproteins with dire consequences (which include cellular glucotoxicity and lipotoxicity) for other organs in the body [10]. A recent systemic review and meta-analysis indicated that the overall prevalence of NAFLD worldwide was estimated to be 32.4% [11]. Overall prevalence of NAFLD was also significantly higher in men than in women [11]. Furthermore, several epidemiological meta-analyses on different human cohorts have indicated that NAFLD is a very strong predictor for all-cause and disease-specific mortality [12]. NAFLD can evolve into nonalcoholic steatohepatitis (NASH) due to a chronic oxidative stress and inflammation. NASH is often associated with ongoing liver damage and can lead to cirrhosis of the liver, hepatocarcinomas, and the need for a liver transplant.

Previous studies on a mouse model with a deletion in its helicase domain (referred as *Wrn^Δhel/Δhel^* mice hereafter) indicated that it phenocopied several WS pathologies. Briefly, *Wrn^Δhel/Δhel^* mice exhibit a distress in the Endoplasmic Reticulum (ER) of their liver at 3–4 months, increased visceral fat and liver inflammation at 4–6 months, dyslipidemia and hyperinsulinemia at 6 months, hepatic lipid accumulation at 7–9 months, cardiac hypertrophy and aortic stenosis at 12 months, neuronal stress at 13 months, and various cancers (including liver cancer) after the age of 15 months [13–16]. Overall, *Wrn^Δhel/Δhel^* mice show a ~17% reduction in median life span compared to wild type mice [16–19].

Recent innovations in proteomic technologies allow the identification and quantification of hundreds of proteins in liver and plasma samples [20]. As such, changes in proteomic profiling are useful to study liver disease progression with time [21]. Preliminary proteomic profiling in the liver of four months old *Wrn^Δhel/Δhel^* males indicated changes in proteins involved in the endoplasmic reticulum stress response as the Wrn mutant protein was predominantly mis-localized in endoplasmic reticulum enriched fractions instead of nuclear fractions [19, 22]. However, the proteome profile has not been examined in older *Wrn^Δhel/Δhel^* mice that exhibit increased dyslipidemia as well as an increase in inflammatory cell aggregations in their liver [18]. In addition, it is unknown whether the proteomic alterations in the liver are the same between *Wrn^Δhel/Δhel^* males and females. In this study, we used label-free Liquid Chromatography-Tandem Mass Spectrometry (LC-MS/MS) to specifically identify proteome profile alterations in the whole liver tissue and the serum of *Wrn^Δhel/Δhel^* mice compared to age-matched wild type animals at four and ten months of age in both males and females. Proteomics analysis at different ages allows us to follow the progressive biological alterations (including histological fat accumulation) in the liver according to age and/or the *Wrn* genotype.

## RESULTS

### Morphological study of liver samples

**Figure 1:**
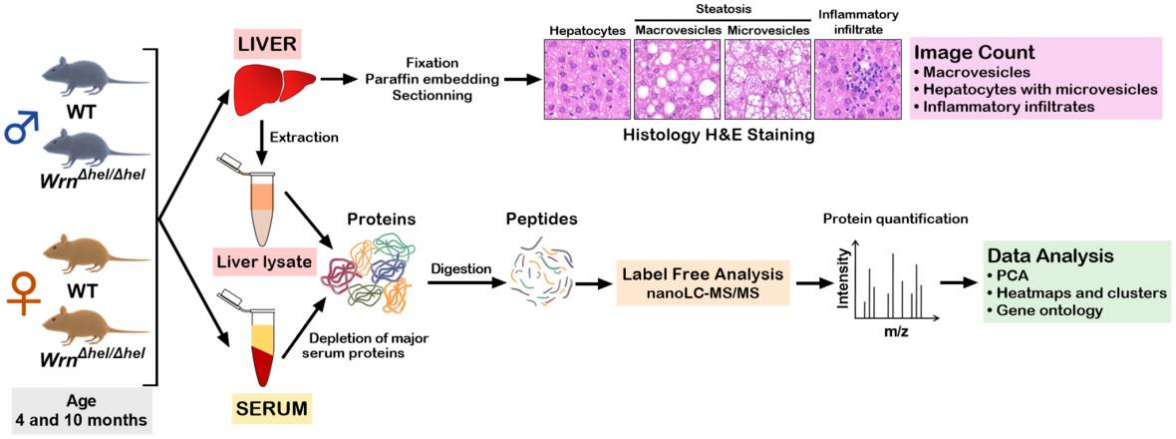
**Experimental design of the study.** Four males and four females at four and ten months of age were euthanized to collect the serum and the whole liver. For each animal, a part of the liver was used for histology analysis and another part was used for protein extraction and mass spectrometry analysis. The whole serum was depleted of abundant proteins before mass spectrometry analyses.

**Figure 2:**
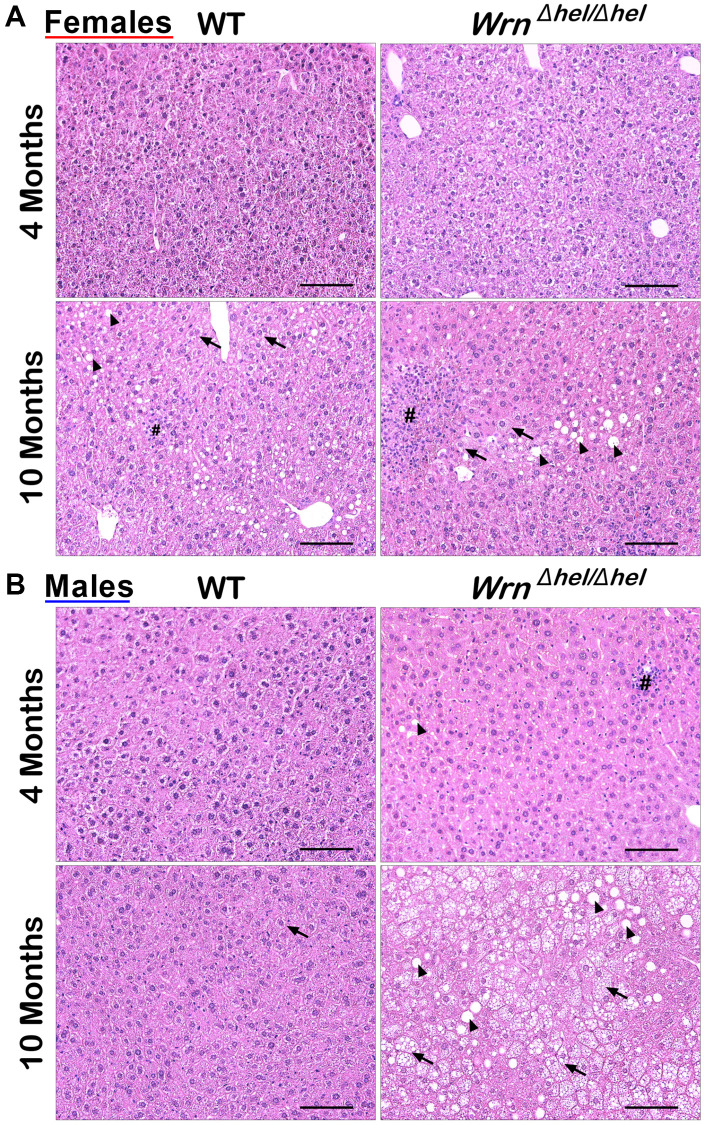
**Representative histology of the liver in wild type and *Wrn^Δhel/Δhel^* mice at four and ten months of age.** (**A**) Representative histological sections from females. (**B**) Representative histological sections from males. All photomicrographs: hematoxylin and eosin; magnification 200X. Scale bars on the images represent 100 μm. # = inflammatory cell aggregation; arrowheads = macrovesicles; arrows = cytoplasmic microvesicles.

**Figure 3:**
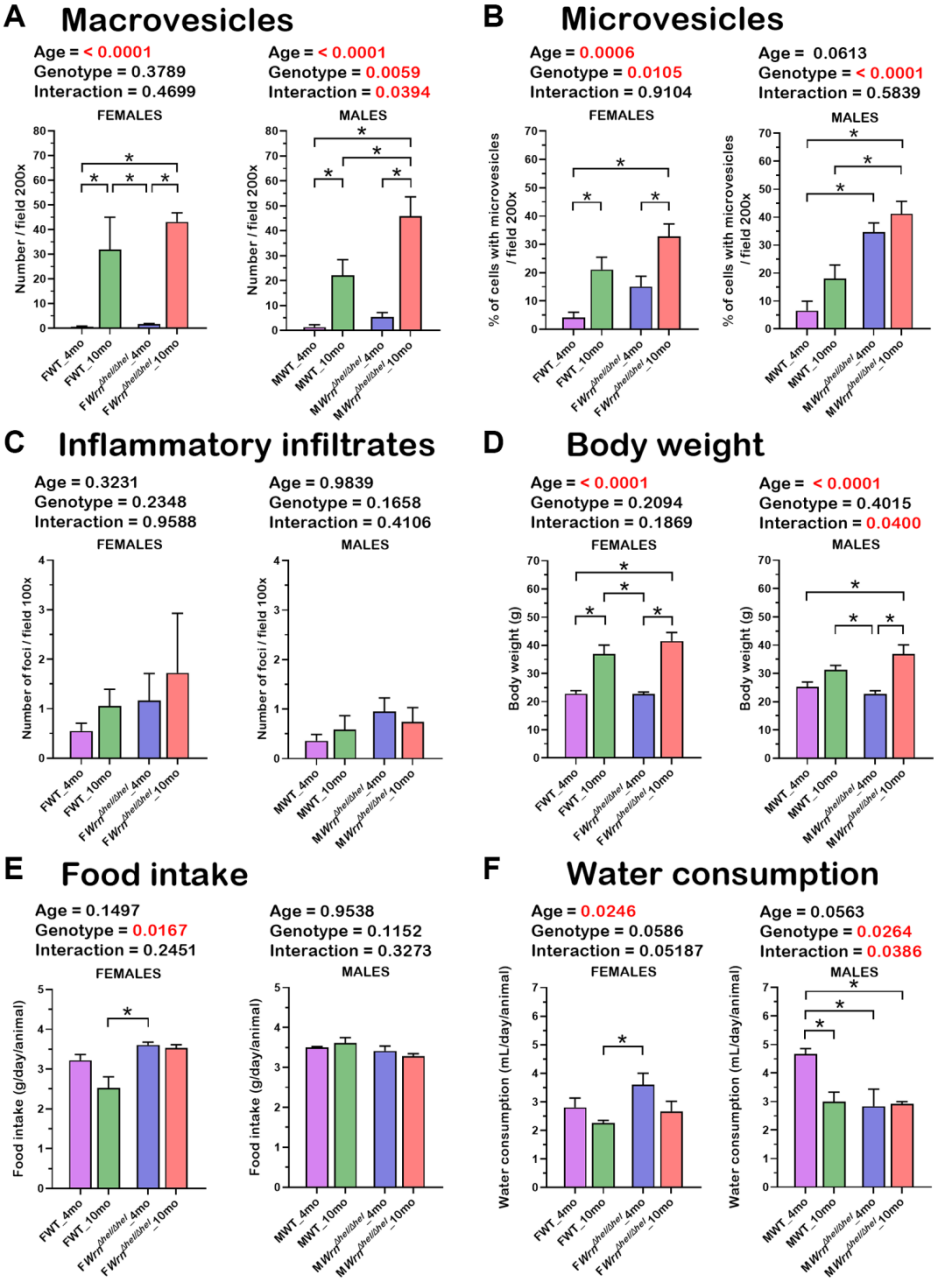
**Histological features of the liver, body weight, food intake, and water consumption of wild type and *Wrn^Δhel/Δhel^* mice at four and ten months of age.** (**A**) Number of macrovesicles. (**B**) Percentage of hepatocytes with enlarged microvesicles. (**C**) Number of inflammatory infiltrates. (**D**) Body weight. (**E**) Food intake. (**F**) Water consumption. (**A**–**D**) *N* = four to six mice per group. (**E**, **F**) *N* = eight to twelve mice per group. All the graphs represent the mean of each group. Bars represent the SEM. Two-way ANOVA *p*-values for age, genotype, and the interaction (age x genotype) are indicated on top of each graph. (Two-way ANOVA followed by Tukey’s multiple comparisons test *p*-value < 0.05 are indicated by ^*^ in the graphs for each comparison).

Previous studies on the liver tissue of *Wrn^Δhel/Δhel^* males indicated an increased stress in the endoplasmic reticulum of young mice (four months old) compared to age-matched wild type males [22] and an increase in inflammatory cell aggregates in the liver of older (> 14 months old) males [18]. In the present study, we analyzed the proteome profiles of the whole liver and the serum of these animals in both males and females at four and ten months of age (young and middle-aged mice) to gain additional information on the biological processes that are altered in the hepatic tissue of *Wrn^Δhel/Δhel^* mice. We focused on four- and ten-months old animals as a previous analysis indicated approximately 11% of *Wrn^Δhel/Δhel^* mice exhibited different types of tumors (including liver tumors) before the age of twelve months [23]. Figure 1 provides the experimental design of the study. There were four different groups for male and female mice. First, hematoxylin and eosin staining were performed on the liver of four to six males and females per group. Hepatocellular steatosis is classified into two types: macrovesicular and microvesicular. In macrovesicular steatosis, a single large fat droplet or smaller well-defined fat droplets occupy the cytoplasm of hepatocytes, pushing the nucleus to the periphery (as exemplified in Figure 1). In microvesicular steatosis, the cytoplasm of hepatocytes is filled with tiny lipid droplets (as depicted in Figure 1). Steatosis in NAFLD is usually macrovesicular; however, microvesicular steatosis may also be present [24]. The presence of microvesicular steatosis is associated with higher grades of steatosis and correlates with more advanced histology of NAFLD [25]. Figure 2 provides representative examples of histological images for wild type and *Wrn^Δhel/Δhel^* females and males at four and ten months of age. The number of macrovesicles (see arrowheads in Figure 2), the percentage of hepatocytes with microvesicles (see arrows in Figure 2), and the number of inflammatory cell aggregates (see # in Figure 2) were counted in all the histological images. Two-way ANOVA followed by Tukey’s multiple comparisons test were performed to determine the effect of age and/or genotype on these histological parameters. Age had a significant effect on the number of macrovesicles in the females (two-way ANOVA *p*-value < 0.0001 in Figure 3A). Indeed, the number of macrovesicles was increased by 59- and 26-fold in wild type and *Wrn^Δhel/Δhel^* females at ten months compared to females at four months of age, respectively (Tukey test ^*^p < 0.05 in the graph of Figure 3A). The number of macrovesicles was significantly increased by 17- and eight-fold in the liver of wild type and *Wrn^Δhel/Δhel^* males at ten months compared to males at four months of age (Tukey test ^*^p < 0.05 in the graph of Figure 3A). However, ten months old *Wrn^Δhel/Δhel^* males had twice as many macrovesicles in their liver than ten months old wild type males (Tukey test ^*^p < 0.05). Accordingly, the two-way ANOVA revealed an interaction between age and genotype on the number of macrovesicles in the liver of males (two-way ANOVA *p*_interaction_ = 0.0394 in Figure 3A).

The percentage of hepatocytes with enlarged cytoplasmic microvesicles was significantly increased by five- and two-fold in the liver of wild type and *Wrn^Δhel/Δhel^* females at ten months compared to wild type females at four months of age, respectively (Tukey test ^*^p < 0.05 in the graph of Figure 3B). Furthermore, the two-way ANOVA indicated that the age and the genotype of the mice affected the percentage of hepatocytes with enlarged cytoplasmic microvesicles in females (*p*_age_ = 0.0006 and *p*_genotype_ = 0.0105). The percentage of hepatocytes with enlarged cytoplasmic microvesicles was significantly increased by five- and six-fold in the liver of *Wrn^Δhel/Δhel^* males at four and ten months of age, respectively, compared to wild type males at four months of age (Tukey test ^*^p < 0.05 in the graph of Figure 3B). The percentage of hepatocytes with microvesicles in ten months old *Wrn^Δhel/Δhel^* males was increased two-fold compared to age-matched wild type males (Tukey test ^*^p < 0.05 in the graph of Figure 3B). The two-way ANOVA indicated that the genotype affected the percentage of hepatocytes with enlarged cytoplasmic microvesicles in males (*p*_genotype_ < 0.0001).

There was an increase in the number of inflammatory foci in the histological sections of *Wrn^Δhel/Δhel^* females compared to age-matched wild type females. However, such increase in the number of inflammatory aggregates was not significant (Figure 3C). The number of inflammatory foci was also increased in all the *Wrn^Δhel/Δhel^* males, but this increase was not statistically significant compared to all the wild type males (Figure 3C).

Mice were weighed and compared between groups. As indicated in Figure 3D, wild type and *Wrn^Δhel/Δhel^* males and females were 20% to 70% heavier at ten months than at four months of age (two-way ANOVA *p*_age_ < 0.0001). Food intake (g of chow per day) was significantly increased in *Wrn^Δhel/Δhel^* females at four months compared to wild type females at ten months by 1.4-fold (Tukey test ^*^p-value < 0.05 in Figure 3E), but it was not different to the other groups of females. No significant difference was observed between all *Wrn^Δhel/Δhel^* males. Finally, water consumption (mL of water per day) was significantly increased between *Wrn^Δhel/Δhel^* females at four months and wild type females at ten months by 1.6-fold (Tukey test ^*^p-value < 0.05 in Figure 3F), but it was not different to the other groups of females. Wild type males drank more water per day at four months than all the other groups of males by ~1.5-fold (Tukey test ^*^p-value < 0.05 in Figure 3F). Thus, food intake and water consumption did not correlate with either the number of macrovesicles or the percentage of hepatocytes with enlarged microvesicles in the different groups of mice.

Finally, we also performed a three-way ANOVA to determine the impact of sex on the number of macrovesicles, the percentage of hepatocytes with microvesicles, the number of inflammatory foci, body weight, food intake, and water consumption (Supplementary Table 1). The percentage of hepatocytes with microvesicles and water consumption were significantly affected by sex (*p*-value < 0.05) in the mouse cohort under study. Despite these sexual differences, the lifespan was similar in both *Wrn^Δhel/Δhel^* males and females (Supplementary Figure 1).

### Liver proteome profiles of wild type and *Wrn^Δhel/Δhel^* mice

**Figure 4:**
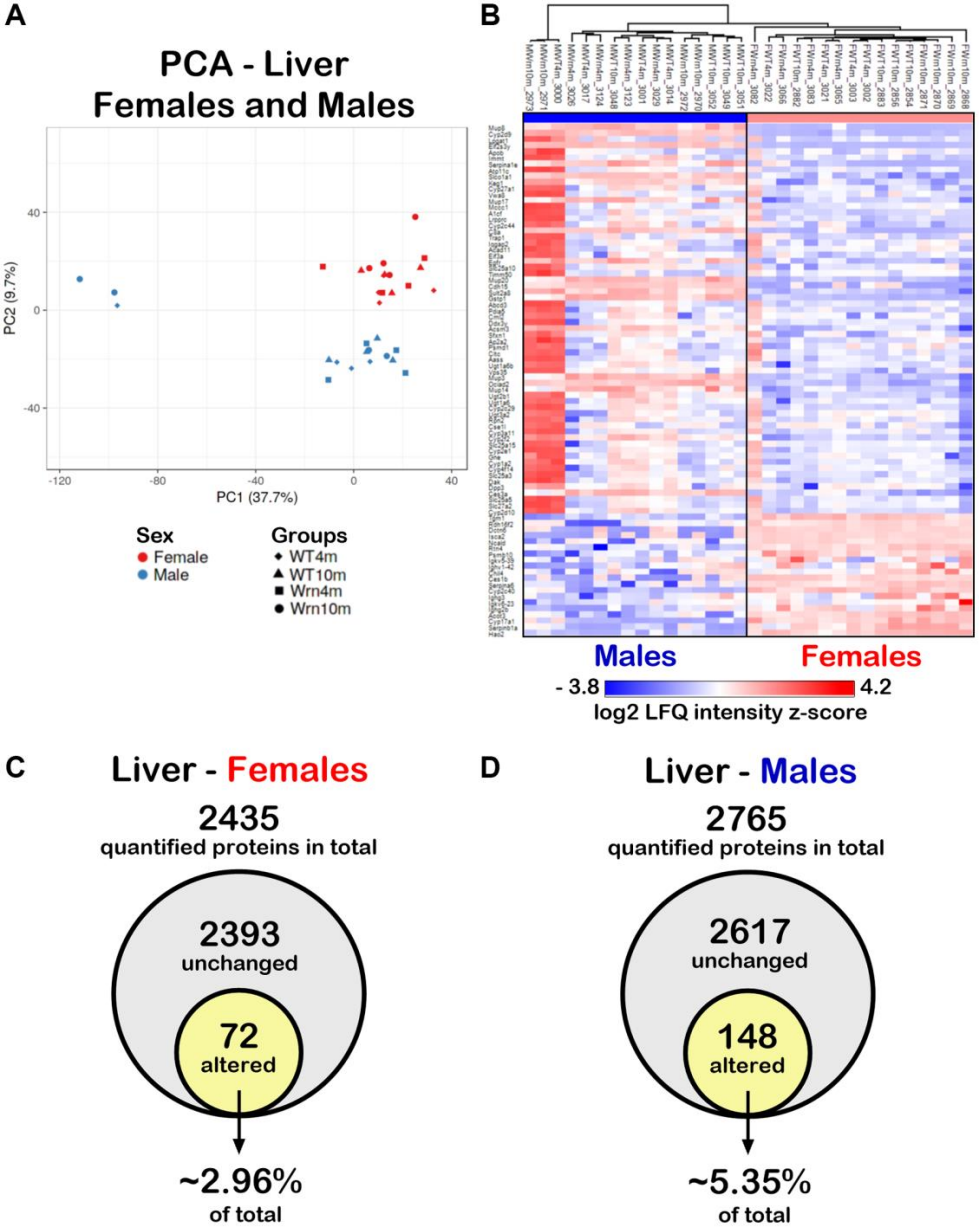
**Impact of sexual dimorphism on the liver proteome profiles of wild type and *Wrn^Δhel/Δhel^* mice at four and ten months of age.** (**A**) Principal component analysis (PCA) graph of all wild type and *Wrn^Δhel/Δhel^* mice at four and ten months of age. (**B**) Heatmap depicting the Z-score value of log base 2 of liver protein (rows) between individual (columns) wild type and *Wrn^Δhel/Δhel^* that significantly separated females and males. (**C**) Venn diagram presenting the number of quantified proteins that were unchanged or altered between the different groups of females. (**D**) Venn diagram presenting the number of quantified proteins that were unchanged or altered between the different groups of males.

To determine whether the liver proteome profiles differed between *Wrn^Δhel/Δhel^* and wild type mice, quantitative proteomic profiling with label-free LC-MS/MS was performed. Four females and four males of each cohort constituting biological replicates were used for the label-free quantification analyses. A total of 3277 proteins in the liver samples were identified (Supplementary Table 2). Principal component analysis (PCA) using the LFQ normalized and imputed data (Supplementary Table 2) were performed. As indicated in Figure 4A, a clear distinction between the liver proteome profiles of females and males was perceptible. To generate a list of proteins that showed significant differences between the females and males, we calculated the mean of the LFQ intensities for each group, the fold change, the *p*-value, and the Z-score for comparison (Supplementary Table 3). The heatmap in Figure 4B shows 84 proteins with significant differences between female and male groups with a *p*-value < 0.05 and a |Z-score| >1.96 and this regardless of the age and genotype of mice. Out of these 84 proteins, 64 and 20 proteins were down and up regulated in the liver of females compared to males, respectively. Enrichments of specific biological processes that were altered in females compared to males were evaluated using the Database for Annotation, Visualization and Integration Discovery (DAVID) tool [26]. The analysis indicated that proteins involved in the xenobiotic metabolic process, exogenous drug catabolic process, organic acid metabolic process, steroid metabolic process, lipid metabolic process, arachidonic acid metabolic process, and cellular response to glucocorticoid stimulus were down regulated in females compared to males (Table 1). The 20 up regulated proteins in the liver of females compared to the liver of males were not associated significantly with a biological process after Bonferroni adjustment.

**Table 1:** Biological processes that significantly differ between females and males in the liver of both wild type and *Wrn^Δhel/Δhel^* groups of mice.

**Biological processes**	**Bonferroni *p*-value**	**Proteins**
**Down regulated in females versus males**		
Xenobiotic metabolic process	6.25E-11	Cyp2c29, Cyp2c23, Cyp2d9, Cyp2e1, Cyp2f2, Ugt1a6b, Gstp1, Ugt1a6, Sult2a8, Cyp2d10
Exogenous drug catabolic process	2.79E-06	Cyp2c29, Cyp2c23, Cyp2d9, Cyp2e1, Cyp2f2, Cyp2d10
Organic acid metabolic process	1.29E-04	Cyp2c29, Cyp2c23, Cyp2d9, Cyp2e1, Cyp2f2, Cyp2d10
Steroid metabolic process	9.81E-04	Cyp1a2, Ugt2b1, Cyp2e1, Apob, Cyp27a1, Sult2a8
Lipid metabolic process	0.0018	Cyp2c29, Slc27a2, Cyp2c23, Acsm3, Cyp2e1, Apob, Gstp1, Cyp27a1, Acad11, Lpgat1, Sult2a8
Arachidonic acid metabolic process	0.0038	Cyp2c29, Cyp2d9, Cyp4f14, Cyp2d10
Cellular response to glucocorticoid stimulus	0.0310	Ugt1a6b, Gstp1, Ugt1a6
**Up regulated in females versus males**		
No significant biological process	−	

### Impact of the Wrn helicase mutation and age on the liver proteome of females and males

To examine the impact of the age and the genotype on the hepatic proteome, we further independently analyzed the mass spectrometry results obtained for the females and males. We quantified 2435 proteins with at least two peptides between the different female groups (Supplementary Table 4) and 2765 proteins with at least two peptides in the different male groups (Supplementary Table 5). To generate lists of proteins that showed significant differences within the four different groups of females or males, we calculated the mean of the LFQ intensities for each group, the fold change, the *p*-value, and the Z-score for each two-by-two comparison. Out of all the quantified proteins in females, 72 proteins differed significantly in at least one of the comparisons between the various female groups with a two-fold change, a *p*-value < 0.05, and a |Z-score| >1.96 (Figure 4C and Supplementary Table 4). Out of all the quantified proteins in males, 148 proteins differed significantly in at least one of the comparisons between the various male groups with a *p*-value < 0.05 and a |Z-score| >1.96 (Figure 4D and Supplementary Table 5). Thus, more hepatic proteins were affected in males (~5.35% of total quantified proteins) than in females (~2.96% of total quantified proteins).

**Figure 5:**
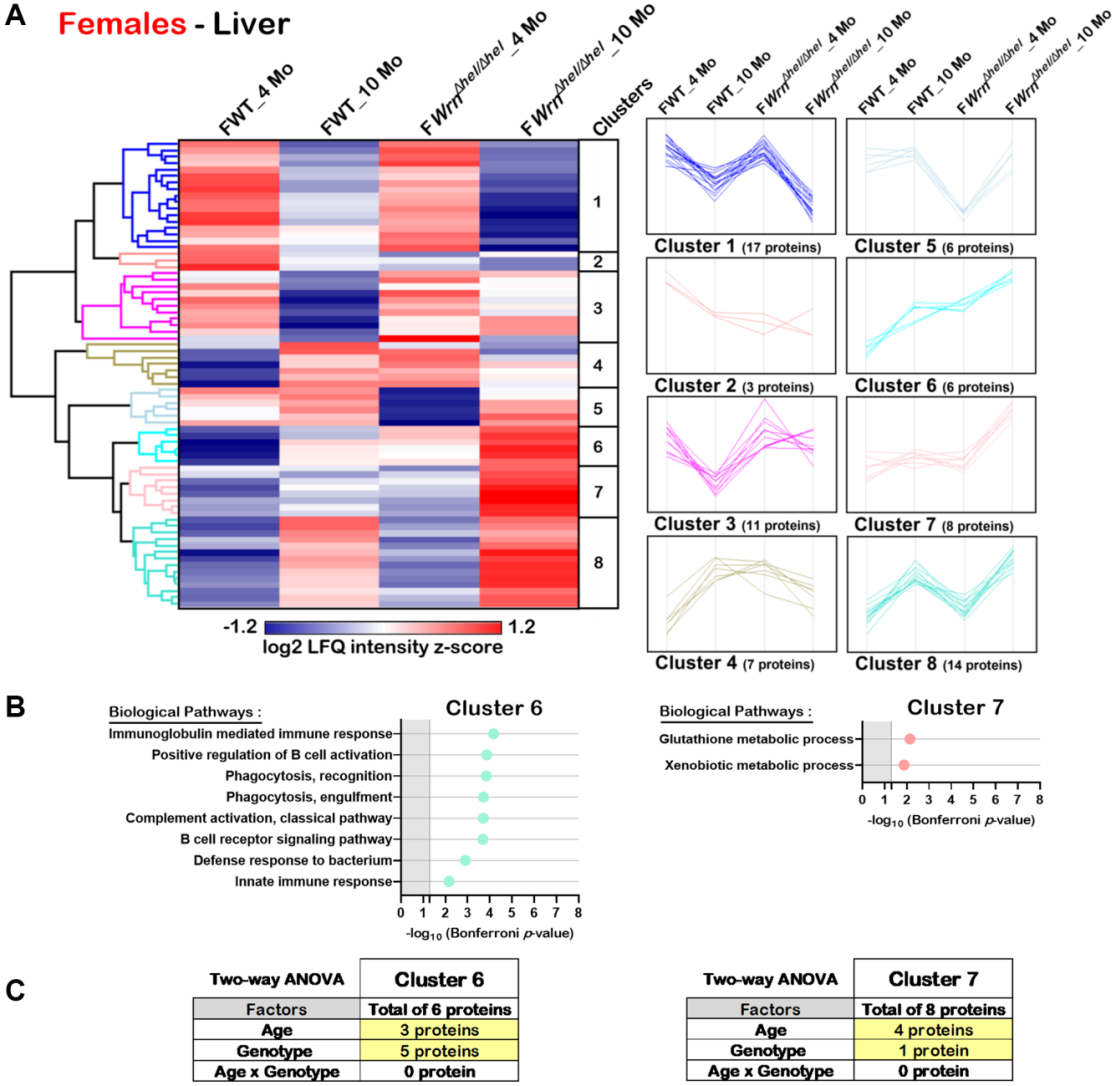
**Significant proteomic changes in the liver of wild type and *Wrn^Δhel/Δhel^* females at four and ten months of age.** (**A**) Hierarchical clustering of label-free quantification (LFQ) intensities of 72 liver proteins that differed significantly in at least one of the comparisons between the various female groups with a two-fold change, a *p*-value < 0.05, and a |Z-score| >1.96. Numbers of proteins and intensity profiles are indicated for each cluster trend plots. (**B**) Gene ontology analysis of Cluster 6 and Cluster 7 that exhibited significant altered biological processes between the different groups of females. (**C**) Two-way ANOVA showing the number of proteins that significantly changed based on the age and/or the genotype of the females in Cluster 6 and Cluster 7.

Hierarchical clustering of the mean of the label-free quantification (LFQ) intensities for the proteins that exhibited a two-fold change between the different groups of females or males with a *p*-value < 0.05 and a |Z-score| >1.96 was performed to gain insights into the biological processes that were altered in *Wrn^Δhel/Δhel^* mice. As shown in Figure 5A, the hierarchical clustering analysis generated by the Perseus software [27] identified eight protein clusters in the liver of females that showed distinct trend profiles with respect to age and/or genotype. Two of these clusters (Cluster 6 and Cluster 7) showed enrichments of specific biological processes based on the DAVID bioinformatics tool [26] with a significant Bonferroni *p*-value. Cluster 6 contained six proteins, four of which are involved in different aspects of the immune system response (Figure 5B). The trend plot of Cluster 6 indicated an increase of their LFQ intensities in *Wrn^Δhel/Δhel^* females at ten months of age compared to all the other female groups (Figure 5A). Interestingly, the LFQ intensities of the *Wrn^Δhel/Δhel^* females at four months of age was similar to the LFQ intensities of the wild type females at ten months of age (Figure 5A). Based on a two-way ANOVA analysis, three proteins from this cluster were significantly different between four- and ten-months females and five proteins were significantly different between wild type and *Wrn^Δhel/Δhel^* females (Figure 5C and Supplementary Table 6). Although three proteins in Cluster 6 were affected by age and genotype, there was no significant interaction between these variables in the liver of females. Cluster 7 contained eight proteins. DAVID analysis indicated an enrichment of glutathione and xenobiotic metabolic processes for this Cluster (Figure 5B). The trend plot of Cluster 7 showed an increase of the LFQ intensities in *Wrn^Δhel/Δhel^* females at ten months of age compared to the other female groups (Figure 5A). Based on a two-way ANOVA analysis, four proteins from this cluster were significantly different between four- and ten-months females and one protein was significantly different between wild type and *Wrn^Δhel/Δhel^* females (Figure 5C and Supplementary Table 6). There was no significant interaction between the age and the genotype in the liver of females for these proteins.

**Figure 6:**
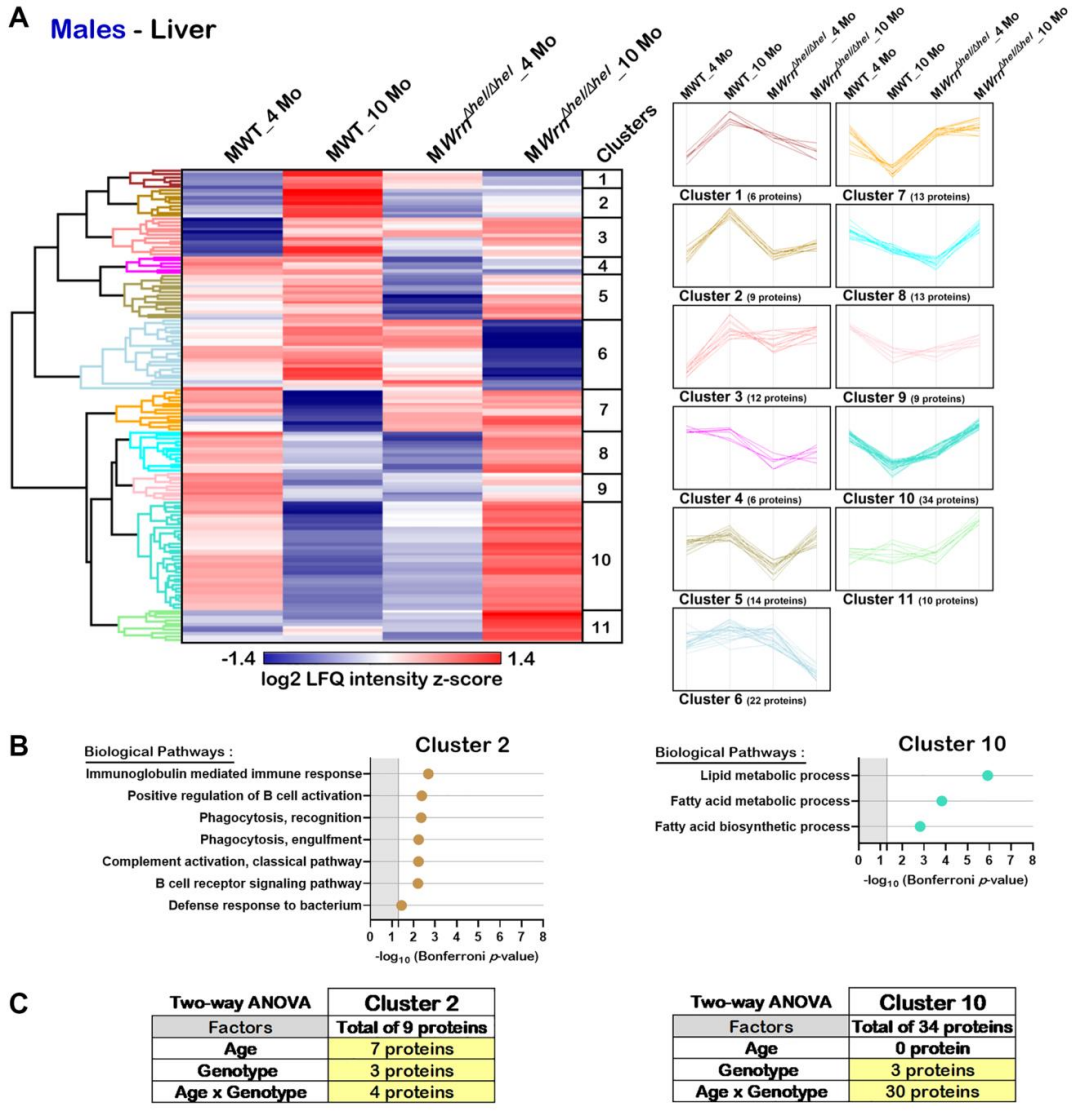
**Significant proteomic changes in the liver of wild type and *Wrn^Δhel/Δhel^* males at four and ten months of age.** (**A**) Hierarchical clustering of label-free quantification (LFQ) intensities of 148 liver proteins that differed significantly in at least one of the comparisons between the various male groups with a two-fold change, a *p*-value < 0.05, and a |Z-score| >1.96. Numbers of proteins and intensity profiles are indicated for each cluster trend plots. (**B**) Gene ontology analysis of Cluster 2 and Cluster 10 that exhibited significant altered biological pathways between the different groups of males. (**C**) Two-way ANOVA showing the number of proteins that significantly changed based on the age and/or the genotype of the males in Cluster 2 and Cluster 10.

Hierarchical clustering analysis identified eleven protein clusters in the liver of males (Figure 6A). DAVID bioinformatics analysis identified two clusters of proteins (Cluster 2 and Cluster 10) associated with enrichments of specific biological processes with a significant Bonferroni *p*-value. Several proteins from Cluster 2 were involved in different aspects of the immune system response (Figure 6B). Various proteins from Cluster 10 were associated with different aspects of lipid or fatty acid metabolic processes (Figure 6B). Cluster 2 was composed of nine proteins with the highest LFQ intensities observed in ten months old wild type males. Based on two-way ANOVA analysis, seven proteins were significantly different between four- and ten-months males and three proteins were significantly different between wild type and *Wrn^Δhel/Δhel^* males (Figure 6C and Supplementary Table 6). There was a significant interaction between age and the *Wrn^Δhel/Δhel^* genotype for four proteins in Cluster 2. The four proteins composing the biological processes of Cluster 2 were mainly affected by an interaction between age and genotype based on two-way ANOVA analysis (Figure 6C and Supplementary Table 6). Cluster 10 was composed of 34 proteins. The LFQ intensities of all these proteins were the highest in the 10 months old *Wrn^Δhel/Δhel^* male group (Figure 6A). Interestingly, the LFQ intensities of these proteins tended to decrease with age in the wild type males but increased with age in the *Wrn^Δhel/Δhel^* males (Figure 6A). Based on two-way ANOVA analysis, there was a significant interaction between the age and the *Wrn^Δhel/Δhel^* genotype for 30 of the 34 proteins in Cluster 10 (Figure 6C and Supplementary Table 6). Thus, the proteins that were involved in lipid and fatty acid metabolic processes showed a significant interaction between age and the *Wrn^Δhel/Δhel^* genotype.

### Serum proteome profiles of wild type and *Wrn^Δhel/Δhel^* mice

**Figure 7:**
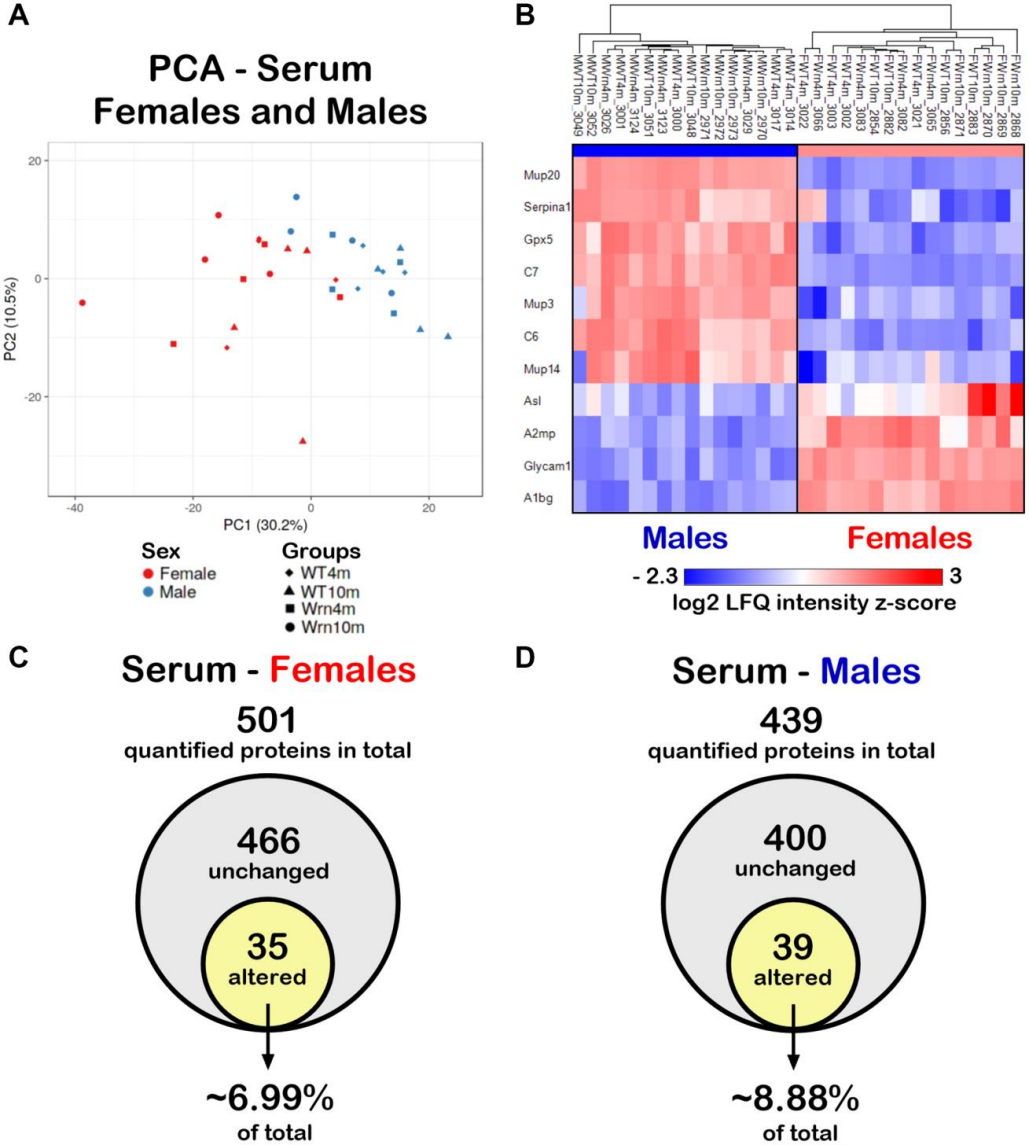
**Impact of sexual dimorphism on the serum proteome profiles of wild type and *Wrn^Δhel/Δhel^* mice at four and ten months of age.** (**A**) Principal component analysis (PCA) graph of all wild type and *Wrn^Δhel/Δhel^* mice at four and ten months of age. (**B**) Heatmap depicting the Z-score value of log base 2 of serum protein (rows) between individual (columns) wild type and *Wrn^Δhel/Δhel^* that significantly separated females and males. (**C**) Venn diagram presenting the number of quantified proteins that were unchanged or altered between the different groups of females. (**D**) Venn diagram presenting the number of quantified proteins that were unchanged or altered between the different groups of males.

Since the liver secretes many abundant systemic proteins including factors involved in various immune functions [20, 28], label-free LC-MS/MS was performed on serum samples from our different mouse groups. A total of 633 proteins in the serum samples could be identified in both females and males (Supplementary Table 7). Principal component analysis (PCA) using LFQ normalized and imputed data (from the Supplementary Table 7) were performed. As indicated in Figure 7A, although some female and male individuals overlapped, overall, a distinction between the serum proteome profiles of females and males was perceptible. To generate a list of proteins that showed significant differences between the females and males, we calculated the mean of the LFQ intensities for each group, the fold change, the *p*-value, and the Z-score for comparison (Supplementary Table 8). The heatmap in Figure 7B shows eleven proteins with significant differences between female and male groups with a *p*-value < 0.05 and a |Z-score| >1.96. Although three major urinary proteins (Mup3, Mup14, and Mup20) and two complement components (C6 and C7) were down regulated in females compared to males, no enrichment of specific biological processes was found to be significantly altered in females compared to males in our cohort using the DAVID bioinformatics tool.

### Impact of the Wrn helicase mutation and age on the serum proteome of females and males

To examine the impact of the age and the genotype on the serum proteome, we further independently analyzed the mass spectrometry results obtained for the females and males. We identified 501 proteins with at least two peptides in the different female groups (Supplementary Table 9) and 439 proteins with at least two peptides in the different male groups (Supplementary Table 10). To generate lists of proteins that showed significant differences between the four different groups of females or males, we calculated the mean of the LFQ intensities for each group, the fold change, the *p*-value, and the Z-score for each two-by-two comparison. For the females, 35 quantified proteins differed significantly in at least one of the groups being compared with a two-fold change, a *p*-value < 0.05, and a |Z-score| >1.96 (Figure 7C and Supplementary Table 9). Out of all the quantified proteins in males, 39 proteins differed significantly in at least one of the comparisons between the various male groups with a two-fold change, a *p*-value < 0.05, and a |Z-score| >1.96 (Figure 7D and Supplementary Table 10). Thus, more serum proteins were affected in males (~8.88% of total quantified proteins) than in females (~6.99% of total quantified proteins).

**Figure 8:**
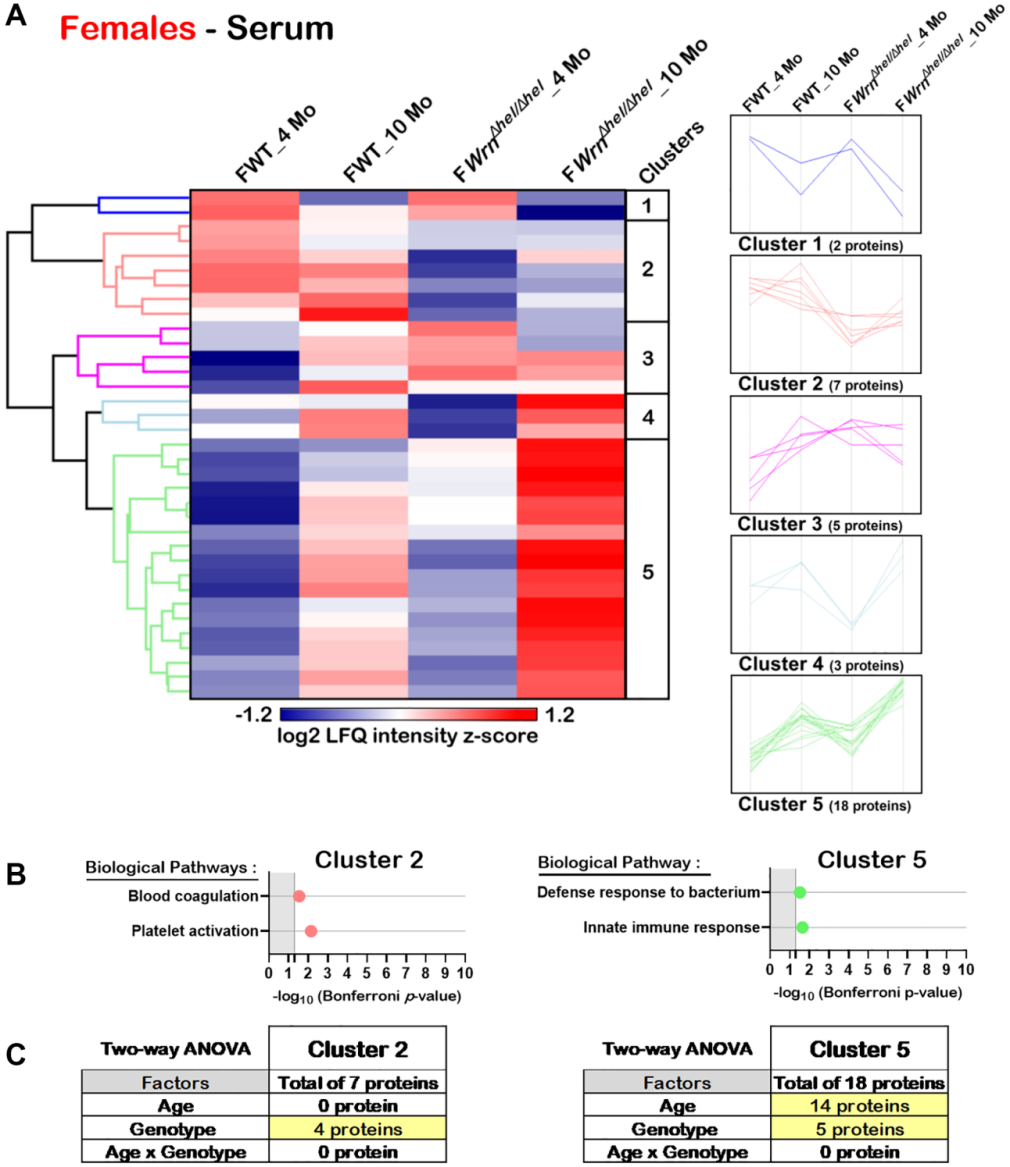
**Significant proteomic changes in the serum of wild type and *Wrn^Δhel/Δhel^* females at four and ten months of age.** (**A**) Hierarchical clustering of label-free quantification (LFQ) intensities of 35 serum proteins that differed significantly in at least one of the comparisons between the various female groups with a two-fold change, a *p*-value < 0.05, and a |Z-score| >1.96. Numbers of proteins and intensity profiles are indicated for each cluster trend plots. (**B**) Gene ontology analysis of Cluster 2 and Cluster 5 that exhibited significant altered biological pathways between the different groups of females. (**C**) Two-way ANOVA showing the number of proteins that significantly changed based on the age and/or the genotype of the females in Cluster 2 and Cluster 5.

Hierarchical clustering of the mean of the label-free quantification (LFQ) intensities for the proteins that exhibited a two-fold change between the different groups of females or males with a *p*-value < 0.05 and a |Z-score| >1.96 was performed to gain insights into the biological processes that were altered in the serum of *Wrn^Δhel/Δhel^* mice. As shown in Figure 8A, the hierarchical clustering analysis identified five protein clusters in the serum of females. Two clusters (Cluster 2 and Cluster 5) showed enrichments of specific biological processes based on gene ontology analysis. Seven proteins were part of Cluster 2 and several of these proteins were involved in platelet activation and blood coagulation processes (Figure 8B). Proteins from these biological processes were mainly affected by the genotype as their abundance was lower in the serum of *Wrn^Δhel/Δhel^* females compared to wild type females (Figure 8A). Based on a two-way ANOVA analysis, four proteins from Cluster 2 were significantly different between wild type and *Wrn^Δhel/Δhel^* females and included proteins associated with blood coagulation and platelet activation (Figure 8C and Supplementary Table 11). Eighteen proteins were part of Cluster 5 and several of these proteins were involved in the innate immune response and/or the bacterial defense response (Figure 8B and Supplementary Table 11). Based on a two-way ANOVA analysis, 14 proteins of Cluster 5 were significantly different between four- and ten-months females and five proteins were significantly different between wild type and *Wrn^Δhel/Δhel^* females (Figure 8C and Supplementary Table 11). Proteins of the immune response process in Cluster 5 were mainly affected by age.

**Figure 9:**
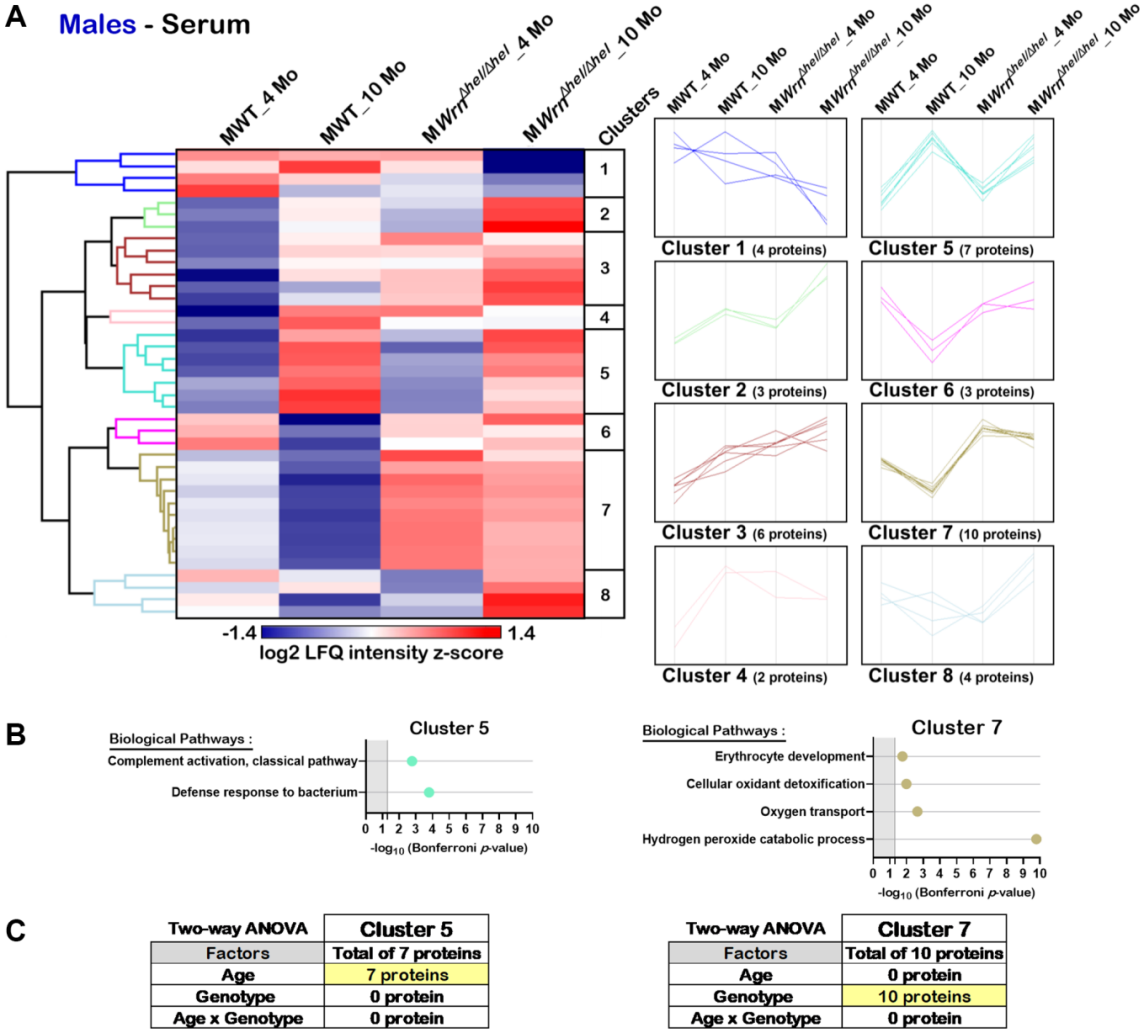
**Significant proteomic changes in the serum of wild type and *Wrn^Δhel/Δhel^* males at four and ten months of age.** (**A**) Hierarchical clustering of label-free quantification (LFQ) intensities of 39 serum proteins that differed significantly in at least one of the comparisons between the various male groups with a two-fold change, a *p*-value < 0.05, and a |Z-score| >1.96. Numbers of proteins and intensity profiles are indicated for each cluster trend plots. (**B**) Gene ontology analysis of Cluster 5 and Cluster 7 that exhibited significant altered biological pathways between the different groups of males. (**C**) Two-way ANOVA showing the number of proteins that significantly changed based on the age and/or the genotype of the males in Cluster 5 and Cluster 7.

Hierarchical clustering analysis identified eight protein clusters in the serum of males (Figure 9A). DAVID bioinformatics analysis identified two clusters of proteins associated with enrichments of specific biological processes (Cluster 5 and Cluster 7). Several proteins from Cluster 5 were involved in the classical complement activation pathway and defense response to bacterium (Figure 9B). Various proteins from Cluster 7 were involved in erythrocyte development, cellular antioxidant detoxification, oxygen transport, and the hydrogen peroxide catalytic process (Figure 9B). Cluster 5 was composed of seven proteins. Based on two-way ANOVA analysis, all these proteins were significantly different between four- and ten-months males (Figure 9C and Supplementary Table 11) and thus the biological processes in Cluster 5 were affected by age. Cluster 7 was composed of ten proteins. Based on two-way ANOVA analysis, all these ten proteins were significantly different between wild type and *Wrn^Δhel/Δhel^* males (Figure 9C and Supplementary Table 11). As such, all the proteins associated with the biological processes identified in Cluster 7 were affected by the genotype as their abundance was higher in all the *Wrn^Δhel/Δhel^* males compared to wild type males regardless of their age (Figure 9B).

### Integration of liver and serum proteomics in wild type and *Wrn^Δhel/Δhel^* mice

**Figure 10:**
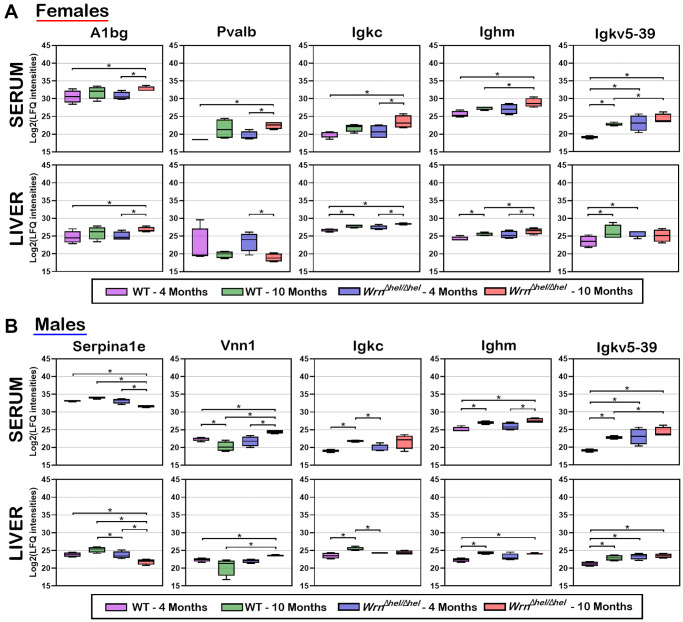
**Box plots of protein LFQ intensities significantly altered between wild type and *Wrn^Δhel/Δhel^* mice at four and ten months of age in both serum and liver samples.** (**A**) Box plots of serum and liver proteins significantly altered in females. (**B**) Box plots of serum and liver proteins significantly altered in males. LFQ intensities are presented as log base 2 values. Significant differences between groups are indicated in each box plot. Proteins were considered significant between the indicated groups with a two-fold change, a limma *p*-value < 0.05, and a |Z-score| >1.96.

To identify proteins whose serum abundance was reflected by alterations in the liver, we integrated the liver and serum proteomes by looking for common features among the datasets in the females (Supplementary Tables 4 and 9) and the males (Supplementary Tables 5 and 10). Figure 10A reveals that five proteins were significantly altered in both the liver and serum when we compared the different groups of females. Alpha-1-B glycoprotein (A1bg) abundance was generally higher in the liver and serum of ten months old *Wrn^Δhel/Δhel^* females than in the other groups of females. Serum parvalbumin (Pvalb) abundance was significantly increased in ten months old *Wrn^Δhel/Δhel^* females compared to four months old wild type and *Wrn^Δhel/Δhel^* females. However, the pattern of Pvalb abundance in the different groups of females was different compared to the serum levels in these same females (^*^p-value < 0.05 in Figure 10A). The abundance of serum immunoglobulin molecules Igkc, Ighm, and Igkv5-39 were similar in the different groups of females. The highest abundance of these molecules was found in the serum of ten months old *Wrn^Δhel/Δhel^* females (^*^p-value < 0.05 in Figure 10A). This pattern of expression was similar in the liver of ten months old *Wrn^Δhel/Δhel^* females for Igkc and Ighm. However, the abundance of Igkv5-39 was the highest in the liver of ten months old wild type females compared to the other groups of females based on mass spectrometry data (^*^p-value < 0.05 in Figure 10A).

Figure 10B shows that five proteins were significantly altered in both the liver and serum when we compared the different groups of males. The abundance of the serine peptidase inhibitor Serpina1e was the lowest in the ten months old *Wrn^Δhel/Δhel^* males compared to the other male groups in both the serum and liver samples (^*^p-value < 0.05 in Figure 10B). In contrast, the abundance of the pantetheinase enzyme Vnn1 (also known as the Vascular non-inflammatory molecule 1 or Vanin 1) was the highest in the ten months old *Wrn^Δhel/Δhel^* males in both the serum and liver samples compared to the other male groups (^*^p-value < 0.05 in Figure 10B). The abundance of the immunoglobulin molecules Igkc was increased in the serum of ten months old wild type and tended to be increased in *Wrn^Δhel/Δhel^* males compared to four months old wild type and *Wrn^Δhel/Δhel^* males in both serum and the liver tissue (^*^p-value < 0.05 in Figure 10B). The abundance of Ighm was higher in the liver and the serum of ten months old *Wrn^Δhel/Δhel^* males compared to wild type males at four months of age. The abundance of Igkv5-39 was lower in four months old wild type males compared to the other groups in both the serum and liver samples. High levels of Igkv5-39 were found in both the serum and liver samples of ten months old *Wrn^Δhel/Δhel^* males (^*^p-value < 0.05 in Figure 10B).

Finally, although the levels of Igkc, Ighm, and Igkv5-39 expressions differed when we compared the liver samples of the various wild type and *Wrn^Δhel/Δhel^* groups of females and males, overall, the highest serum levels of Igkc, Ighm, and Igkv5-39 proteins were observed in both the ten months old *Wrn^Δhel/Δhel^* females and males (Figure 10A, 10B).

### Serum immune cytokine levels in wild type and *Wrn^Δhel/Δhel^* mice with age

**Figure 11:**
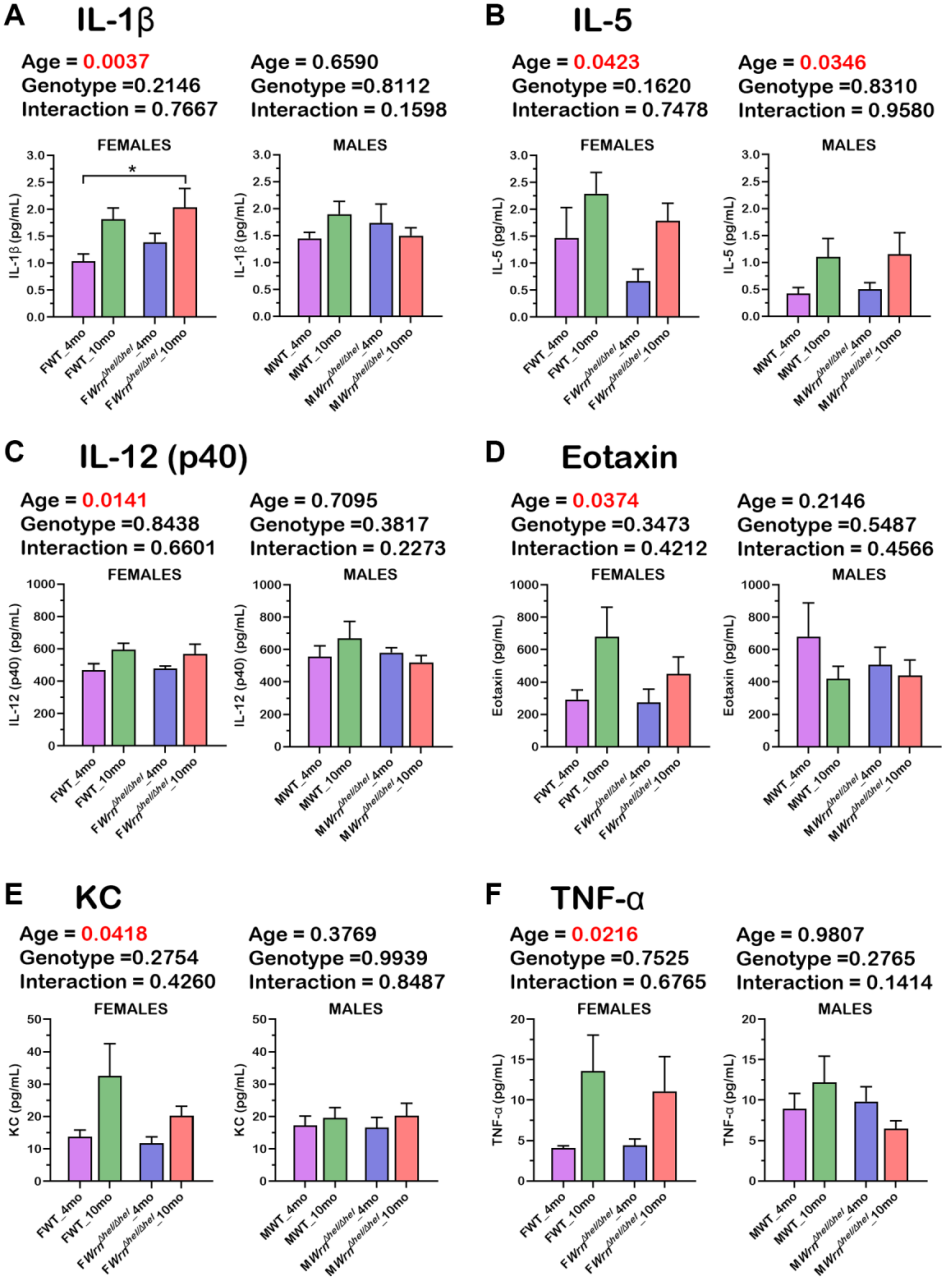
**Serum levels of six cytokines significantly altered in the various wild type and *Wrn^Δhel/Δhel^* female and male groups.** (**A**) IL-1ß. (**B**) IL-5. (**C**) IL-12 (p40 subunit). (**D**) Eotaxin. (**E**) KC. (**F**) TNF-α. All the graphs represent the mean of each group. Bars represent the SEM. Two-way ANOVA *p*-values for age, genotype, and the interaction (age x genotype) are indicated on top of each graph. Two-way ANOVA followed by Tukey’s multiple comparisons test *p*-value < 0.05 are indicated by ^*^ in the graphs for each comparison. (*N* = 9–12 males or females per group).

Since cytokines of the immune system were not detected in the serum of our mouse cohort by mass spectrometry analysis, we measured 23 serum cytokines using a multiplex assay kit (as described in Materials and Methods). The histograms in the Figure 11A–11F show that six cytokines exhibited a significant difference between four- and ten-months old females independently of the *Wrn^Δhel/Δhel^* genotype (two-way ANOVA *p*-values < 0.05). These cytokines included IL-1ß, IL-5, IL-12 (p40 subunit), Eotaxin, KC, and TNF-α. The fold increase in ten months old females compared to four months old females varied from 1.2-fold (for IL-12 (p40 subunit) in Figure 11C) to 3.3-fold (for TNF-α in Figure 11F). Additional Tukey test on these cytokines indicated a significant two-fold increase in *Wrn^Δhel/Δhel^* females at ten months of age compared to wild type females at four months of age (Figure 11A). IL-5 was the only cytokine that showed a significant difference based on age in the males (Figure 11B) independently of the *Wrn^Δhel/Δhel^* genotype (two-way ANOVA *p*-values < 0.05). No significant difference was observed between the various groups of *Wrn^Δhel/Δhel^* and wild type males for all the other analyzed cytokines. Finally, three-way ANOVA did not show a sex dependent effect on the serum levels of the analyzed cytokines.

### Senescence in the liver of wild type and *Wrn^Δhel/Δhel^* mice with age

**Figure 12:**
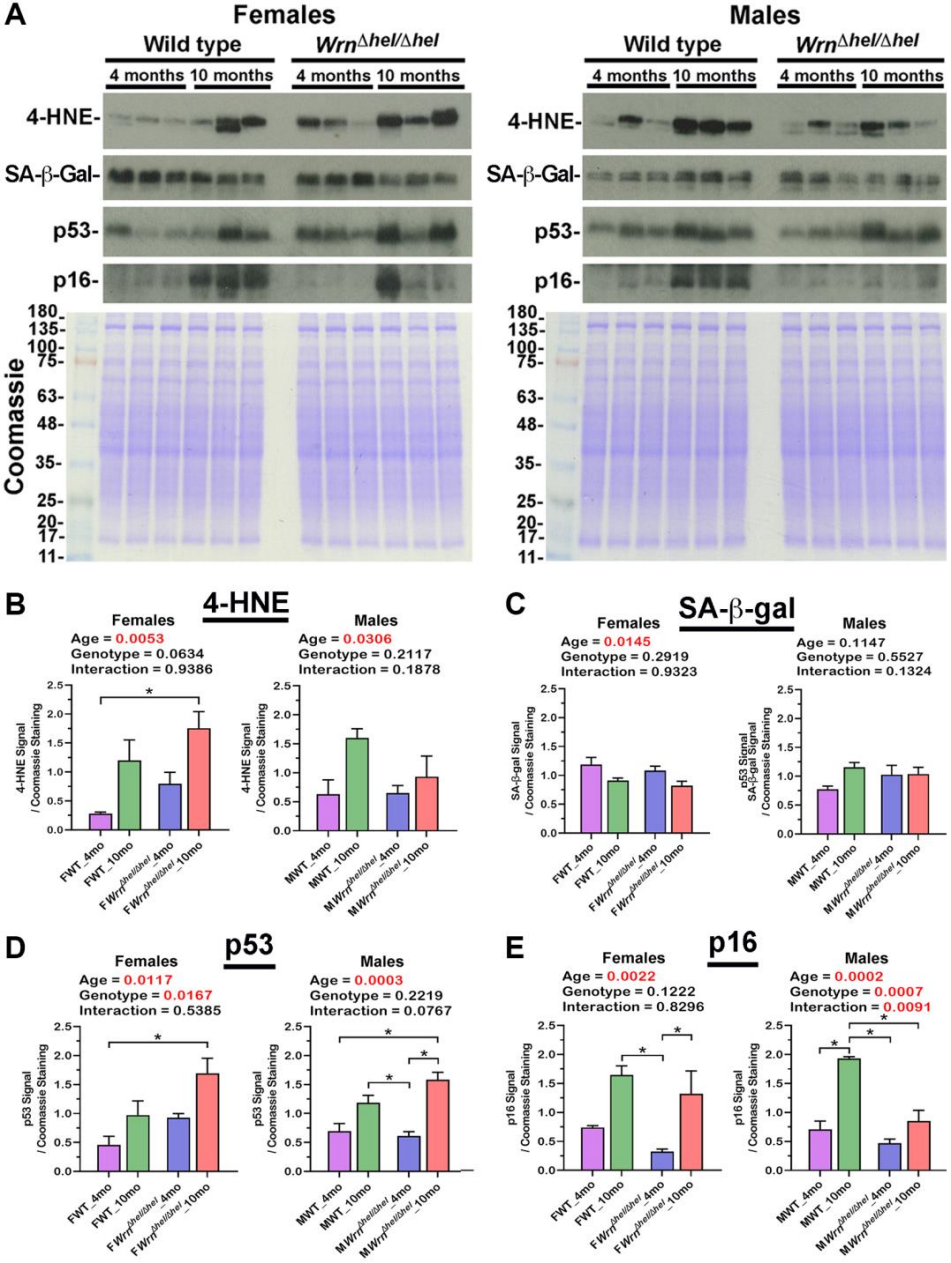
**Immunoblot analyses of markers of lipid peroxidation and senescence in the liver of wild type and *Wrn^Δhel/Δhel^* mice.** (**A**) Examples of western blots showing the levels of 4-hydroxynonenal (4-HNE)-protein adducts, senescence-associated ß-galactosidase (SA-ß-gal), p53, and p16 proteins in both female and male groups. The lower panels show Coomassie staining of the gels. The numbers on the left of the Coomassie represent the molecular weights in kDa. (**B**) Ratio of the 4-HNE signal over Coomassie staining from the western blots. (**C**) Ratio of the SA-ß-gal signal over Coomassie staining from the western blots. (**D**) Ratio of the p53 signal over Coomassie staining from the western blots. (**E**) Ratio of the p16 signal over Coomassie staining from the western blots. All the graphs represent the mean of each group. Bars represent the SEM. Two-way ANOVA *p*-values for age, genotype, and the interaction (age x genotype) are indicated on top of each graph. Two-way ANOVA followed by Tukey’s multiple comparisons test *p*-value < 0.05 are indicated by ^*^ in the graphs for each comparison. (*N* = 3 males or females per group).

Abnormal accumulation of lipids in the liver can increase lipid peroxidation, protein damage, and stress induced senescence of hepatocytes [29]. Western blot analyses were thus performed to assess the levels of 4-hydroxynonenal (4-HNE)-protein adducts and the levels of senescence markers such as senescence-associated ß-galactosidase (SA-ß-gal), p53, and p16 proteins in both female and male groups. Examples of western blots for these markers are shown in Figure 12A. The levels of 4-HNE-protein adducts were increased by four and two-fold in the liver of ten months old *Wrn^Δhel/Δhel^* and wild type females compared to four months old *Wrn^Δhel/Δhel^* and wild type females, respectively. Two-way ANOVA indicated that age affected 4-HNE-protein adducts in females (*p*_age_ = 0.0053). Additional Tukey test indicated a significant difference between four months old wild type and ten months old *Wrn^Δhel/Δhel^* females (Tukey test ^*^p < 0.05 in the graph of Figure 12B). Two-way ANOVA on the different male groups also indicated that age affected 4-HNE-protein adducts (*p*_age_ = 0.0306). However, the difference between four and ten months old wild type and *Wrn^Δhel/Δhel^* males were less than 2.5 and 1.4-fold, respectively (Figure 12B).

Although two-way ANOVA indicated that the age affected the levels of SA-ß-gal in the liver of all the females, additional Tukey tests did not reveal a significant difference between the various female groups (Figure 12C). Interestingly, the western blot analysis of SA-ß-gal confirmed the data obtained with the mass spectrometry analysis (Supplementary Figure 2). The LFQ intensities of Glb1 (the gene coding for SA-ß-gal) were lower in older wild type and *Wrn^Δhel/Δhel^* females (two-way ANOVA *p*_age_ = 0.0083). However, unlike the western blot analysis, additional Tukey tests on the LFQ data showed a significant difference between the four months old wild type and ten months old *Wrn^Δhel/Δhel^* females (Tukey test ^*^p < 0.05 in the graph of Supplementary Figure 2). SA-ß-gal levels in the four months old wild type males tended to be ~1.3-fold lower than the other male groups. However, two-way ANOVA did not show significant differences between the various male groups (Figure 12C).

The protein level of p53 was significantly increased by 3.7-fold in the liver of ten months old *Wrn^Δhel/Δhel^* females compared to four months old wild type females (Tukey test ^*^p < 0.05 in the graph of Figure 12D). Furthermore, the two-way ANOVA indicated that the age and the genotype of the females affected p53 levels (*p*_age_ = 0.0117 and *p*_genotype_ = 0.0167). The p53 level was significantly increased by 2.3 and 2.5-fold in the liver of ten months old *Wrn^Δhel/Δhel^* males compared to wild type and *Wrn^Δhel/Δhel^* males at four months of age, respectively (Tukey test ^*^p < 0.05 in the graph of Figure 12D). The two-way ANOVA indicated that the age of the males affected p53 levels (*p*_age_ = 0.0003).

The protein level of p16 was significantly increased by five- and four-fold in the liver of ten months old wild type and *Wrn^Δhel/Δhel^* females, respectively, compared to *Wrn^Δhel/Δhel^* at four months of age (Tukey test ^*^p < 0.05 in the graph of Figure 12E). Furthermore, the two-way ANOVA indicated that the age of the females affected the p16 levels in the liver (*p*_age_ = 0.0022). The level of p16 was increased by more than two-fold in the ten months old wild type males compared to the other male groups (Tukey test ^*^p < 0.05 in the graph of Figure 12E). Although p16 levels were increased by 1.8-fold in the liver of ten months old *Wrn^Δhel/Δhel^* compared to four months old *Wrn^Δhel/Δhe^* males, this difference was not significant. Nevertheless, the two-way ANOVA indicated that p16 was affected by both the age and the genotype of the males (*p*_age_ = 0.0002 and *p*_genotype_ = 0.0007).

Overall, although the p53 and p16 proteins were not detected in our mass spectrometry analyses, the immunoblot experiments indicated that the levels of 4-HNE, p53, and p16 were at least affected by age in the liver of both male and female wild type and *Wrn^Δhel/Δhel^* mice.

## DISCUSSION

The major goal of this study was to look at murine hepatic proteomic profiles at two different time points and determine the impact of a mutation in the *Wrn* gene product with age in the liver of mice. Mice were fed ad libitum with normal chow and were not treated with any drugs or chemicals that can induce liver lesions. Histological examination of the liver in the wild type and *Wrn^Δhel/Δhel^* mice at four and ten months of age indicated an increase in macrovesicular and microvesicular steatosis with age. However, there is no evidence that food intake or water consumption correlated with the number of macrovesicles or the percentage of hepatocytes with enlarged microvesicles in the different groups of mice. This agrees with reports showing that aging is associated with marked liver fat accumulation in mice [30, 31].

It has been reported in two different mouse models of fatty liver disease that males exhibit a more severe histological fatty liver phenotype than females in adults during their reproductive age. However, this sex difference is no more observed in older (9 months of age) mice experiencing a significant decline in the reproductive status of their life [32]. Such age-dependent sex differences in mice are consistent with those observed in human NAFLD [32]. In the present study, ad libitum fed wild type and *Wrn^Δhel/Δhel^* mice involved ten months old animals, thus implicating female mice that passed their prime for reproduction [33]. Accordingly, the number of macrovesicles and the percentage of hepatocytes with microvesicles were increased in both males and females by ten months of age in wild type and *Wrn^Δhel/Δhel^* mice. Nevertheless, some differences could be detected between *Wrn^Δhel/Δhel^* males and females. First, the percentage of hepatocytes with microvesicles in *Wrn^Δhel/Δhel^* males was twice the percentage recorded for *Wrn^Δhel/Δhel^* females at four months of age. Importantly, microvesicular steatosis is associated with a higher grade hepatosteatosis in humans [25]. Second, principal component analyses (PCA) on both the whole liver and serum proteome profiles showed a sexual dimorphism regardless of age and *Wrn* genotype. Thirdly, our proteomic data showed that more proteins were significantly altered between the different groups (ages and/or genotypes) in the liver of males than in the liver of females (5.35% and 2.96% of the quantified proteins in males and females, respectively). Finally, gene ontology analysis on our proteomic data indicated an enrichment of proteins involved in lipid and fatty acid metabolisms in *Wrn^Δhel/Δhel^* males unlike females. Such results with *Wrn^Δhel/Δhel^* mice agree with transcriptomic analyses on different models of diet induced NAFLD indicating that males exhibited greater changes in differentially expressed genes than females by the age of seven months [34]. Furthermore, hierarchical clustering followed by a two-way ANOVA indicated an interaction between the age and the genotype parameters for proteins involved in lipid and fatty acid metabolism in the liver of males. Thus, we infer that the Wrn mutation exacerbates the age-related fat accumulation in the liver in agreement with the significant increase of microvesicular steatosis observed even in the younger males compared to age-matched wild type counterparts.

The proteins that showed a significant difference between females and males in our cohorts were mainly involved in the cellular response to glucocorticoid stimuli as well as xenobiotic, exogenous drug, organic acid, steroid, lipid, and arachidonic acid metabolisms. Such a finding agrees with previous published reviews on the liver being a sexually dimorphic organ [35, 36]. Although the establishment of the molecular sexual dimorphism in the liver remains to be fully characterized, the impact of sexual hormones and the differential expression pattern of growth hormones in females and males are under intense investigations [36]. Furthermore, four out of the eleven proteins that were significantly altered between the serum samples of all the females and males were part of the immune complement cascade. This observation agrees with a study reporting that systemic complement activity in female mice is reduced due to androgen regulation of hepatic complement synthesis [37].

Hierarchical clustering of the proteins that showed significant level differences between either group of mice followed by gene ontology analysis permitted the identification of biological pathways that were defined by the age and/or the genotype of the mice. The major observation in the liver of females is an increase of proteins involved in different aspects of the immune response system that is mainly affected by their genotype. The highest LFQ intensities were found in ten months old *Wrn^Δhel/Δhel^* females. Although the same biological processes were affected in the liver of males, the highest LFQ intensities were not found in the ten months old *Wrn^Δhel/Δhel^* males. This result could be due to the higher interindividual variations observed within the males than within the female groups as indicated by the PCA graph of the liver samples. Nevertheless, despite the stringent Bonferroni correction applied on the gene ontology analysis, there was an increase in the immune response with age in the liver of both females and males consistent with an augmented state of chronic inflammation as one of the hallmarks of aging [1]. Although not statistically significant, there was an increased trend in the number of inflammatory infiltrates in the histological sections of *Wrn^Δhel/Δhel^* liver tissues compared to those of wild type tissues. One limitation of the histological analysis is that microscopic sections did not provide an extent of the inflammatory infiltrations within the whole liver tissues. Isolation and characterization of immune cells infiltrating the hepatic tissue in older mice with appropriate immune markers will be required to identify the hematopoietic cell types involved. Note that we did not observe significant alterations in proteins specifically associated with other cell types than hepatocytes such as Kupffer cells or stellate cells in males or females. Specific cell type isolation or spatial proteomics experiments will also be required to identify tissue areas of distinct protein expression in the liver of these mice.

In the serum, proteins involved in bacterial defense response, the innate immune response or the complement activation pathway were increased with age in both females and males. In addition to proteins involved with the immune response system, hierarchical clustering of the serum proteomic data indicated a decrease in proteins involved in platelet activation and blood coagulation in the serum of *Wrn^Δhel/Δhel^* females at both four and ten months of age. Thus, platelet activation and blood coagulation were mainly affected by the genotype of the females. Such processes were not significantly affected in *Wrn^Δhel/Δhel^* males. It has been reported that platelet activation decreases with old age in female mice [38]. It will be interesting to see whether blood coagulation and platelet activation are more affected in women with Werner syndrome.

*Wrn^Δhel/Δhel^* males exhibited an increase of proteins involved in erythrocyte development, cellular antioxidant detoxification, and hydrogen peroxide catalytic processes at four and ten months of age. Such processes were not significantly affected in the serum of *Wrn^Δhel/Δhel^* females. The increase of cellular antioxidant response in the serum of *Wrn^Δhel/Δhel^* males agrees with the increase of lipid and fatty acid metabolic processes in the liver of the same males at ten months of age. Although the lipid peroxidation detected by the levels of 4-HNE-protein adducts in the liver was increased in both older females and males, the extent of this increase was less important in *Wrn^Δhel/Δhel^* males compared to females (Figure 12B). This result agrees with the increased cellular antioxidant detoxification and hydrogen peroxide catalytic responses observed in the serum of *Wrn^Δhel/Δhel^* males. Interestingly, it has been reported in diet-induced mouse models of fatty liver diseases that males exhibited an increase in the relative abundance of markers of oxidative stress response compared to females [34].

When both the liver and serum proteomic profiles were integrated in the analysis, three immunoglobulin variants were common in the liver and serum of both females and males and included Igkc, Ighm, and Igkv5-39. Although the abundance of Igkc, Ighm, and Igkv5-39 differed when we compared the liver with the serum samples of the various wild type and *Wrn^Δhel/Δhel^* groups of females and males, overall, the highest serum levels of Igkc, Ighm, and Igkv5-39 proteins were observed in both the ten months old *Wrn^Δhel/Δhel^* females and males (Figure 10). The results are also consistent with the low systemic but chronic inflammation observed in Werner syndrome patients [6, 39]. More interestingly, increased expression of the immunoglobulin variants IGKC and IGHM has been associated with NAFLD as well as liver fibrosis and cirrhosis in human subjects [40–43]. Although analyses on the activation status of the immune cells in *Wrn^Δhel/Δhel^* mice compared to age-matched wild type mice are required, the measurements of several serum cytokines in the mouse cohorts have indicated an increase of IL-5 in both females and males at ten months of age compared to four months old groups of mice. IL-5 is known to regulate the innate and acquired immune response and it induces the proliferation and activation of B-cells leading to the secretion of immunoglobulins [44]. The levels of IL-5 in the different groups of mice in our cohort concord with the serum immunoglobulins levels shown in the immune response protein clusters of the heatmaps in the Figure 8A and 9A for females and males, respectively. Of note, a cross-sectional study has reported that serum IL-5 levels are augmented with obesity in human subjects [45]. Although thorough proteomic analyses of the adipose tissues in our mouse cohorts will be required, the IL-5 results in Figure 11B agree with the observed increase in body weight of *Wrn^Δhel/Δhel^* and wild type mice at ten months of age in both females and males (Figure 3D). The serum cytokine analysis also revealed significant increases of IL-1ß and TNF-α in wild type and *Wrn^Δhel/Δhel^* females at ten months of age (Figure 11). These cytokines are known to be increased in human subjects with NAFLD and are considered biomarkers for NASH as well [46]. IL-1ß and TNF-α were not significantly increased in *Wrn^Δhel/Δhel^* males at ten months of age compared to the other male groups of our cohort. This could be due to higher interindividual variations in the males compared to females as depicted in the liver proteome profiles of males (PCA in Figure 4A).

In addition to the increase of specific immunoglobulin variants, the serum and whole liver proteomes showed an increase of A1bg in the females (especially in the *Wrn^Δhel/Δhel^* female group) at ten months of age. This was not observed in the males based on our stringent criteria. A1bg is a small plasma glycoprotein of unknown function. The protein shows sequence similarity to the variable regions of some immunoglobulin supergene family member proteins and maybe involved in inflammatory response. More importantly, a Selected Reaction Monitoring (SRM) targeted proteomics study has shown an increase of serum A1BG during fibrosis to cirrhosis progression in human patients enrolled in the “Hepatitis C Antiviral Long-term Treatment against Cirrhosis Trial” [47]. Furthermore, human serum A1BG levels performed well in distinguishing patients from healthy controls (using the Area Under the Receiver-Operating-Characteristic curve as a criterion) [47]. More recently, a proteomic study on patients with cirrhosis also showed a significant increase of blood A1BG levels compared to healthy subjects [42]. It has been suggested that A1BG may potentially prove useful for chronic liver diseases [47].

In contrast to females, *Wrn^Δhel/Δhel^* males showed an increase of the Vnn1 protein (also known as Vanin 1) at ten months of age in both the whole liver and serum (Figure 10). Vnn1 is an enzyme primarily involved in vitamin B5 recycling, which is an important precursor for the synthesis of Coenzyme A [48]. Several studies have indicated that Vnn1 controls chronic inflammatory response and negatively regulates oxidative stress-induced intrinsic apoptotic signaling pathway [49, 50]. It has been suggested that Vanin 1 promotes inflammation as a knockout of this gene in mice protects them against tissue inflammation through a glutathione-mediated resistance to oxidative stress [51]. Various integrative omics studies have indicated that Vanin 1 is increased in several mouse models of fatty liver diseases in both the plasma and liver of males [20, 34]. Importantly, Vanin 1 has been shown to be increased in human NAFLD [52] and could be a potential biomarker of fatty liver disease progression [20, 34].

*Wrn^Δhel/Δhel^* males also exhibited a significant decrease in Serpina1e abundance in both the serum and liver tissue at ten months of age (Figure 10). Liver and serum Serpina1e have been shown to be decreased in various mouse models of fatty liver diseases [53, 54]. Serpina1e is the anti-protease alpha 1-antitrypsin that protects tissues from proteolytic degradation by neutrophil elastases. It is secreted in the blood stream mainly by the liver [54]. Serpina1e is especially decreased in the liver by diets rich in saturated fatty acids in mice [53]. Interestingly, a previous lipidomic study indicated that *Wrn^Δhel/Δhel^* males had an abnormal increase of long chain saturated and monounsaturated lipids in their serum compared to wild type mice [19], which may affect the levels of liver and serum Serpina1e in *Wrn^Δhel/Δhel^* males. Importantly, several studies have reported that the activity of the human orthologue SERPINA1 is decreased in NAFLD and NASH and could be potentially used as a predictive serum biomarker of chronic liver diseases [55, 56].

A previous study on a mouse model of NAFLD reported that in addition to inflammation, an abnormal accumulation of lipids in the liver increases stress induced senescence of the hepatocytes [29]. Western blot and mass spectrometry analyses did not show significant differences in SA-ß-gal protein levels between groups of female or male wild type and *Wrn^Δhel/Δhel^* mice (based on two-way ANOVA followed by Tukey tests). It is possible that ten months old mice (mid-aged animals) were still too young to show significant differences in hepatic SA-ß-gal protein levels between wild type and *Wrn^Δhel/Δhel^* mice. In contrast, the levels of other proteins potentially associated with cellular senescence such as p53 and p16 were mainly affected by the age of the wild type and *Wrn^Δhel/Δhel^* mice in both females and males based on two-way ANOVA. The analysis also indicated that the genotype significantly affected p53 levels only in the females and p16 levels only in the males. Such data agrees with a literature review article suggesting sex difference in the cellular senescence process [57].

In summary, our detailed proteomic profiles study of wild type and *Wrn^Δhel/Δhel^* mice at four and ten months of age revealed a sexual dimorphism in the liver tissue and the serum of these animals. Interestingly, although both females and males exhibited macrovesicular and microvesicular steatosis, proteins implicated in lipid and fatty acid metabolic processes were specifically increased in the liver of ten months old *Wrn^Δhel/Δhel^* males. We infer that a mutation in the Wrn enzyme exacerbated these processes with age. Serum proteins related to oxidant detoxification processes were also increased in *Wrn^Δhel/Δhel^* males regardless of age. Finally, several proteins that were altered in both the liver and serum of middle-aged *Wrn^Δhel/Δhel^* males and females are also associated with different aspects of chronic liver diseases (from NAFLD to NASH and cirrhosis) in human subjects based on the literature. Further investigation on the combination of these protein alterations through machine learning will be required to assess their relevance to NAFLD status and progression in human subjects. Future thorough examination of NAFLD in Werner syndrome patients will indicate whether similar biological processes are also affected in such patients and whether they exhibit sexual dimorphisms.

## MATERIALS AND METHODS

### Animals and maintenance

Mice lacking part of the helicase domain of the Wrn gene were generated by homologous recombination, as described previously [58]. This study was performed on wild type and *Wrn^Δhel/Δhel^* homozygous animals on C57BL/6NHsd genetic background (Harlan Laboratories, Frederick, MD, USA) and was carried out in strict accordance with the recommendations from the Canadian Council on Animal Care in science. The protocol was approved by the Committee on the Ethics and Protection of Animal of Laval University (Permit Number: CHU-22-1039). Mice were housed in microisolator cages (containing a top filter) at 22 ± 2°C with 40–50% humidity and a 12-h light-dark cycle (light cycle: 07:00-19:00 hours) in the Centre Hospitalier de l’Université Laval animal facility. All mice were fed ad libitum with Teklad Global (cat. # 2018; Madison, WI, USA) 18% protein rodent diet (5% fat). Mice were fasted overnight and body weights were measured before liver collection. Liver was collected at 10:00 am the next day after blood harvesting by cardiac puncture and final exsanguination under general anesthesia (with 3% isoflurane) at the age of four and ten months. Liver samples were frozen at −80°C. Blood was allowed to clot for one hour on ice and spun on a bench top centrifuge at 16,000 *g* for 15 min. Collected serum was frozen in aliquots at −80°C until execution of analyses.

### Liver histology

Fresh liver tissue was fixed using 10% formalin (Fisher Scientific, Pittsburgh, PA, USA). Hematoxylin and eosin (H&E) staining was performed on paraffin embedded sections using standard methods [59]. Four to five pictures for each animal were counted to identify histologic features of fatty liver and included macrovesicular steatosis and percentage of hepatocytes with microvesicles [60]. One-way ANOVA followed by post-hoc Tukey tests were performed using GraphPad Prism version 9.5.0 for Windows (GraphPad Software, San Diego, CA, USA). All reported values in the histograms are mean ± standard error of the mean (SEM). Differences were considered significant at a *p*-value < 0.05.

### Whole liver protein extraction

Tissue protein extraction was carried out in lysis buffer containing 50 mM Tris-HCl (pH 7.5), 150 mM NaCl, 1% NP-40, 0.2% SDS, 1% sodium deoxycholate, 1 mM phenylmethylsulfonylfluoride, complete protease inhibitor cocktail and phosphatase inhibitor cocktail PhoSTOP™ (Roche Applied Science, Indianapolis, IN, USA). After tissue homogenization and sonication, samples were centrifuged at 16,000 *g* for 15 min thrice. Protein concentration was determined by the Bradford protein assay (Bio-Rad, Mississauga, ON, Canada). Samples were frozen at −80°C until mass spectrometry analysis.

### Preparation of sample for label-free liquid chromatography-tandem mass spectrometry

Preparation of protein samples for mass spectrometry analysis were performed as described in previous studies by Aumailley et al. [61, 62]. Briefly, each liver protein sample was precipitated with 5 volumes of acetone overnight. The protein pellet was recovered by centrifugation at 16,000 *g* for 15 min and resuspended in 200 μL of 1% DOC/50 mM ammonium bicarbonate buffer. A Bradford protein assay was performed to estimate protein concentration. Ten μg of proteins from whole liver lysate were digested with trypsin. Briefly, proteins were first reduced with 0.2 mM dithiothreitol for 30 min at 37°C and alkylated with 0.8 mM iodoacetamide for 30 min at 37°C. Samples were then incubated with trypsin (trypsin:protein ; 1:50) at 37°C overnight. The reaction was stopped by addition of 1% trifluoroacetic acid (TFA), 0.5% acetic acid, and 0.5% acetonitrile then centrifuged for 5 min at 16,000 *g*. The peptides obtained were then desalted using C18 stagetip.

For serum, the most abundant proteins were depleted from 25 μl of serum sample with the Multiple Affinity Removal Spin Cartridge Mouse-3 kit according to the manufacturer’s instructions (Agilent, Santa Clara, CA, USA). Ten μg of depleted serum were resuspended in 50 mM ammonium bicarbonate/1.0% sodium deoxycholate, heated 5 min at 95°C, reduced with 0.2 mM dithiothreitol for 30 min at 37°C, then alkylated with 0.8 mM iodoacetamide at 37°C for 30 min. Proteins were finally digested with trypsin (Promega, Madison, WI, USA) (ratio 1:50) overnight at 37°C. Samples were desalted using stage-tip [63]. Protein concentrations were measured with a peptide nanodrop assay.

### Label-free liquid chromatography-tandem mass spectrometry analysis

Two μg of each serum sample or 1.25 μg of each liver sample was analyzed by nanoLC-MS/MS using a Dionex UltiMate 3000 nanoRSLC chromatography system (Thermo Fisher Scientific, Waltham, MA, USA) connected to an Orbitrap Fusion mass spectrometer (Thermo Fisher Scientific) equipped with a nanoelectrospray ion source. Peptides were trapped at 20 μL/min in loading solvent (2% acetonitrile, 0.05% TFA) on a 5 mm x 300 μm C18 pepmap cartridge pre-column (Thermo Fisher Scientific) for 5 min. Then, the pre-column was switched online with Pepmap Acclaim column (Thermo Fisher Scientific) 50 cm × 75 μm internal diameter separation column and the peptides were eluted with a linear gradient from 5–40% solvent B (A: 0.1% formic acid, B: 80% acetonitrile, 0.1% formic acid) in 90 min, at 300 nL/min for a total run time of 120 min. Mass spectra were acquired using a data dependent acquisition mode using Thermo XCalibur software version 4.1.50. Full scan mass spectra (350 to 1800 m/z) were acquired in the orbitrap using an AGC target of 4e5, a maximum injection time of 50 ms, and a resolution of 120,000. Internal calibration using lock mass on the m/z 445.12003 siloxane ion was used. Each MS scan was followed by acquisition of fragmentation MS/MS spectra of the most intense ions for a total cycle time of 3 s (top speed mode). The selected ions were isolated using the quadrupole analyzer in a window of 1.6 m/z and fragmented by Higher energy Collision-induced Dissociation (HCD) with 35% of collision energy. The resulting fragments were detected by the linear ion trap in rapid scan rate with an AGC target of 1e4 and a maximum injection time of 50 ms. Dynamic exclusion of previously fragmented peptides was set for a period of 30 s and a tolerance of 10 ppm.

### Database searching and label free quantification (LFQ)

Searches in databases and quantification of spectra were performed as previously described [62]. Briefly, spectra were searched against the Uniprot Ref *Mus musculus* database (July 2020 release/63807 entries) using the Andromeda module of MaxQuant software v. 1.6.10.43 [64]. Trypsin/P enzyme parameter was selected with two possible missed cleavages. Carbamidomethylation of cysteins was set as fixed modification while methionine oxidation, protein N-terminal acetylation and hydroxyproline were set as variable modifications for the global search. Mass search tolerance was 5 ppm and 0.5 Da for MS and MS/MS, respectively. For protein validation, a maximum False Discovery Rate of 1% at peptide and protein level was used based on a target/decoy search. MaxQuant was also used for Label Free Quantification. The ‘match between runs’ option was used with 20 min value as alignment time window and 0.7 min as match time window. Only unique and razor peptides were used for quantification. Normalisation (LFQ intensities) was performed by MaxQuant.

### LFQ data post-processing and statistical analysis

RStudio 1.2.5019 was used for data post-processing. In the case of protein intensity values that were missing, values were replaced by a noise value corresponding to 1% percentile of the normalised value for each condition. A protein was considered as quantifiable only if at least three intensity values in the three replicates of one of the two conditions being compared were present and if two peptides or more were identified for this protein. A ratio of LFQ intensity means, a Z-score, and a limma *p*-value were calculated. To be significantly differentially expressed, a protein needed to have a *p*-value less than 0.05 and a Z-score lower than -1.96 (under-expressed) or higher than 1.96 (over-expressed). Principal Component Analysis (PCA) was performed with ClustVis [65].

Hierarchical clustering analysis was performed using Perseus (MaxQuant, v2.0.7.0, Martinsried, Germany) [27]. Briefly, normalized and imputed LFQ intensities were first log base 2 transformed. Then two-way ANOVA was calculated to examine the influence of two categorical factors (age and genotype of mice). Values were z-scored without any grouping. Row distance calculated using the Euclidean algorithm with average-linkage settings without constraint were used to obtain the final hierarchical clustering results. Profile plots were finally generated for each determined cluster of proteins.

Enrichments of specific biological processes were evaluated using the Database for Annotation, Visualization and Integration Discovery (DAVID) tool [26]. Gene ontology terms were considered significant with a Bonferroni *p*-value < 0.05 as described before for proteomics data [22, 61, 62].

Box plots were generated with log base 2 of the LFQ intensities with GraphPad Prism. Each box extends from the 25th to the 75th percentiles. The line inside each box represents the median. The whiskers go down to the smallest value and up to the largest value for each group of mice.

### Cytokine measurements in serum samples

The following cytokines were assessed in the serum (diluted 1:4) using the multiplex kit from Bio-Rad (catalogue number MD60009RDPD): IL-1α, IL-1β, IL-2, IL3, IL-4, IL-5, IL-6, IL-9, IL-10, IL-12 (p40 subunit), IL-12 (p70 subunit), IL-13, IL-17A, Eotaxin, G-CSF, GM-CSF, IFN-γ, KC, MCP-1, MIP-1α, MIP-1β, Rantes, and TNF-α. All measurements were performed on a Bio-Plex^®^ 200 system (with Bio-Plex Manager™ software version 6.0) from Bio-Rad Laboratories Canada Ltd. (Mississauga, ON, Canada).

### Immunoblotting analysis

Western blotting experiments on whole liver protein samples from our different groups were performed as previously described [62]. The separated proteins on the PVDF membranes were detected using the following antibodies: a mouse monoclonal antibody against 4-hydroxynonenal adducts of histidine residues (4-HNE (clone # 198960) #MAB3249) from R&D Systems (Toronto, ON, Canada), a mouse monoclonal antibody against p53 tumor suppressor protein (p53 (1C12) #2524) from Cell Signaling (Whitby, ON, Canada), and rabbit monoclonal antibodies against p16 INK4A protein (p16 INK4A (E5F3Y) #29271) and senescence associated-β-galactosidase (β-Galactosidase (E2U2I) #27198S) from Cell Signaling (Whitby, ON, Canada). Coomassie blue dye was used to stain proteins in SDS-PAGE gels and allowed the quantification of the loaded proteins per well with the ImageJ Software (https://imagej.net/ij/index.html).

### Data availability

All mass spectrometry data (raw files and MaxQuant search result files) are publicly available at MassIVE (https://massive.ucsd.edu/) with the identifier MSV000091632.

## Supplementary Materials

Supplementary Figures

Supplementary Table 1

Supplementary Table 2

Supplementary Table 3

Supplementary Table 4

Supplementary Table 5

Supplementary Table 6

Supplementary Table 7

Supplementary Table 8

Supplementary Table 9

Supplementary Table 10

Supplementary Table 11
