# Supplementary material for: Integrated liver and serum proteomics uncover sexual dimorphism and alteration of several immune response proteins in an aging Werner syndrome mouse model
Source: Aging (Albany NY). 2024 May 24;16(10):8417–45. doi: 10.18632/aging.205866 (PMC11164518; doi:10.18632/aging.205866)
Supplement: Supplementary Figures [file aging-16-205866-s001.pdf]

SUPPLEMENTARY FIGURES

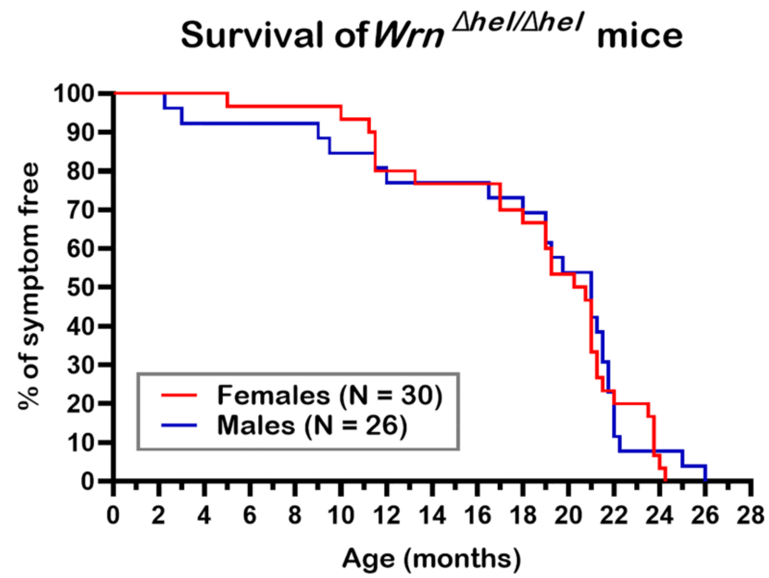

Supplementary Figure 1. Impact of the *Wrn*$^{\Delta hel/\Delta hel}$ genotype on the lifespan of males versus females. The graph represents the percentage of disease-free animals with age. The number of mice in each group is indicated.

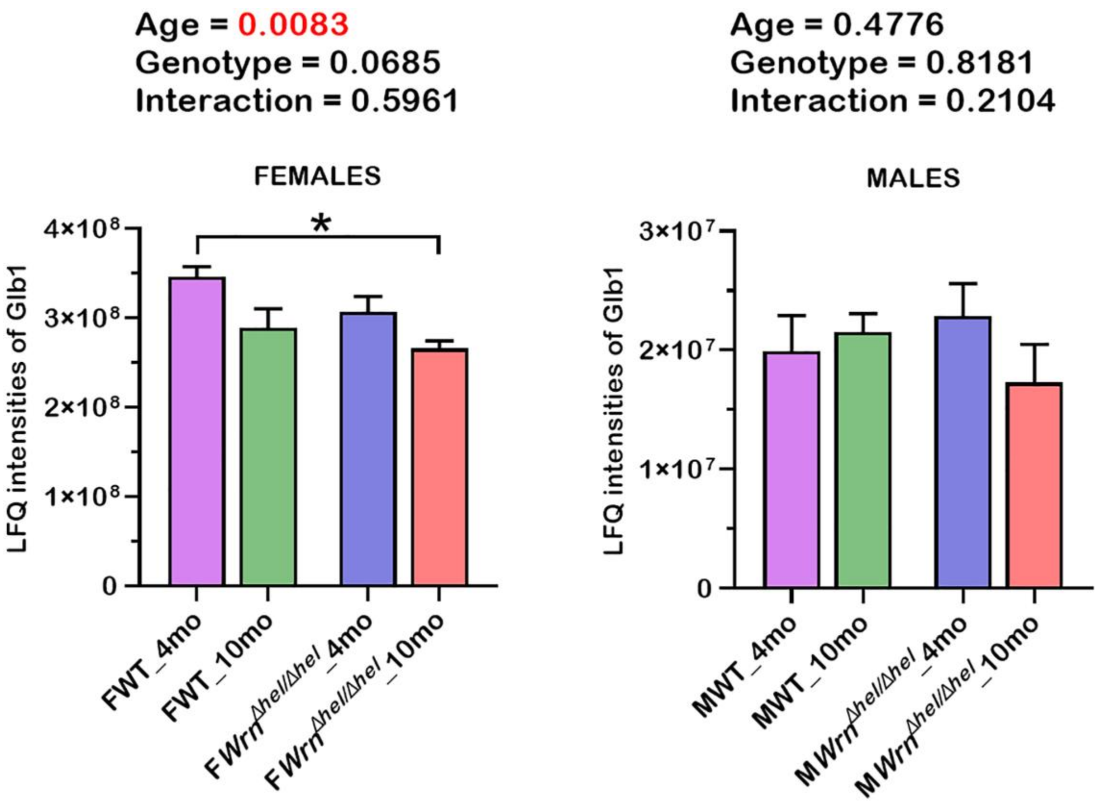

Supplementary Figure 2. Label Free Quantification intensities of Glb1 gene product encoding the senescence-associated ß-galactosidase enzyme. All the graphs represent the mean LFQ intensities of each group. Bars represent the SEM. Two-way ANOVA *p*-values for age, genotype, and the interaction (age x genotype) are indicated on top of each graph. Two-way ANOVA followed by Tukey's multiple comparisons test *p*-value < 0.05 are indicated by * in the graphs for each comparison.
