# Supplementary material for: Integrated liver and serum proteomics uncover sexual dimorphism and alteration of several immune response proteins in an aging Werner syndrome mouse model
Source: Aging (Albany NY). 2024 May 24;16(10):8417–45. doi: 10.18632/aging.205866 (PMC11164518; doi:10.18632/aging.205866)
Supplement: Supplementary Table 1 [file aging-16-205866-s002.pdf]

SUPPLEMENTARY TABLE

Supplementary Table 1. Three-way ANOVA to determine the impact of sex on the number of macrovesicles, the percentage of hepatocytes with microvesicles, the number of inflammatory foci, body weight, food intake, and water consumption.

	Three-way ANOVA					
**Source of variation**	**Macrovesicles**	**Microvesicles**	**Inflammation**	**Body weight**	**Food intake**	**Water consumption**
Sex	0.8858	0.0266	0.1413	0.1418	0.1797	0.0322
Genotype	0.0263	<0.0001	0.1094	0.1418	0.1658	0.4497
Age	<0.0001	0.0001	0.3925	<0.0001	0.2511	0.0028
